# Supplementary material for: Proteo-transcriptomic reprogramming and resource reallocation define the aging mammalian brain
Source: bioRxiv. 2025 Aug 19:2025.08.14.669896. Preprint. [Version 1] doi: 10.1101/2025.08.14.669896 (PMC12393264; doi:10.1101/2025.08.14.669896)
Supplement: 1 [file NIHPP2025.08.14.669896v1-supplement-1.pdf]

bioRxiv preprint doi: https://doi.org/10.1101/2025.08.14.669896; this version posted August 19, 2025. The copyright holder for this preprint (which was not certified by peer review) is the author/funder, who has granted bioRxiv a license to display the preprint in perpetuity. It is made available under aCC-BY 4.0 International license.

Supplementary Figures

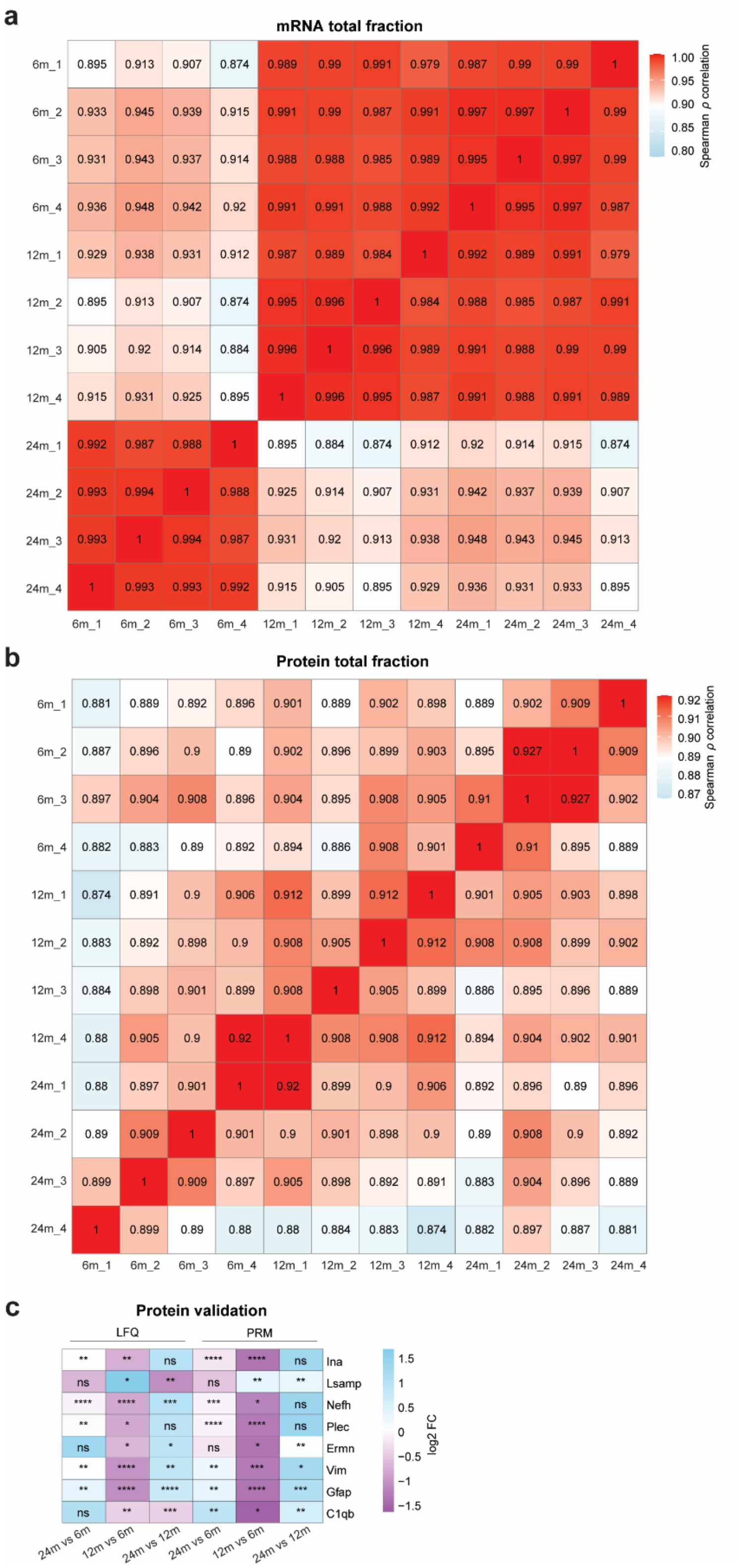

Extended Data Figure 1: mRNA and protein correlation and validation. (a) Heatmap depicting Spearman ρ correlation across biological replicates for the mRNA-total fraction**. (b)** Heatmap for the protein-total fraction, with red indicating high correlation and sky blue indicating low correlation. **(c)** Validation of proteins using label-free quantification (LFQ) and Parallel Reaction Monitoring (PRM) analyses, where sky blue represents increased expression with aging and medium orchid denotes decreased expression with aging.

bioRxiv preprint doi: https://doi.org/10.1101/2025.08.14.669896; this version posted August 19, 2025. The copyright holder for this preprint (which was not certified by peer review) is the author/funder, who has granted bioRxiv a license to display the preprint in perpetuity. It is made available under aCC-BY 4.0 International license.

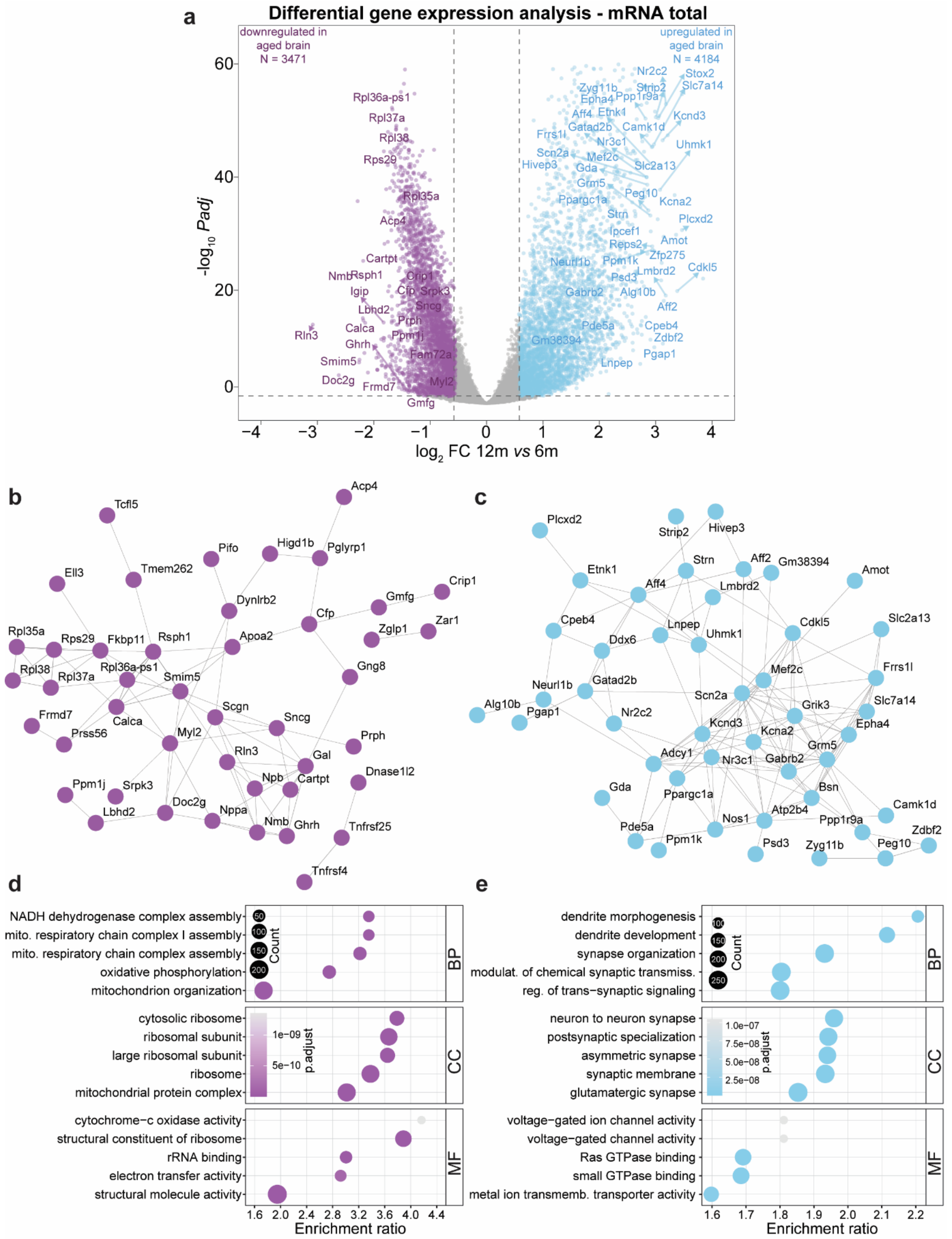

Extended Data Figure 2: Gene expression changes in the aging mouse brain for 12m vs 6m in mRNA-total fraction. (**a**) Volcano plot displaying the differentially expressed genes for 12m vs 6m. Inset: dot plot for biological replicate variability calculated using PCA, where sky blue represents increased expression with aging and medium orchid denotes decreased expression with aging. (**b, c**) STRING representation for the top 50 most (**b**) down- or (**c**)upregulated genes in 12m brain (respectively medium orchid and light blue). (**d, e**) GO-ORA of genes that are significantly down- or upregulated in 12m (*padj* ≤ 0.05, $|\log_2 FC| \geq 0.58$**.**

bioRxiv preprint doi: https://doi.org/10.1101/2025.08.14.669896; this version posted August 19, 2025. The copyright holder for this preprint (which was not certified by peer review) is the author/funder, who has granted bioRxiv a license to display the preprint in perpetuity. It is made available under aCC-BY 4.0 International license.

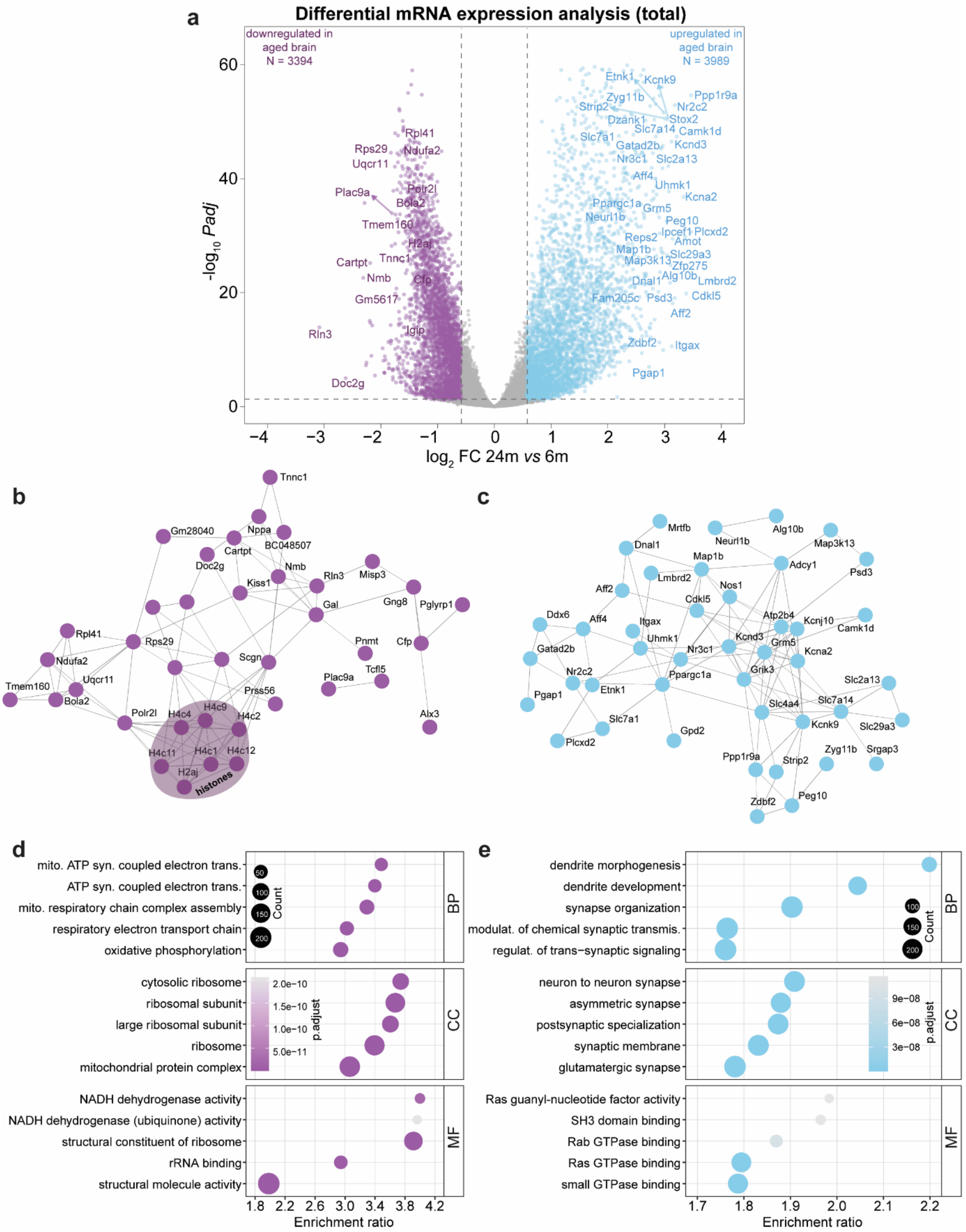

1456 **Extended Data Figure 3: Gene expression changes in the aging mouse brain for 24m vs 6m in mRNA-total**
1457 **fraction.** (**a**) Volcano plot displaying the differentially expressed genes for 24m vs 6m. Inset: dot plot for biological
1458 replicate variability calculated using PCA, where sky blue represents increased expression with aging and medium
1459 orchid denotes decreased expression with aging. (**b, c**) STRING representation for the top 50 most (**b**) down- or
1460 (**c**)upregulated genes in 24m brain (respectively medium orchid and light blue). (**d, e**) GO-ORA of genes that are
1461 significantly down- or upregulated in 24m (*padj* ≤ 0.05, |$\log_2$FC| ≥ 0.58).
1462

bioRxiv preprint doi: https://doi.org/10.1101/2025.08.14.669896; this version posted August 19, 2025. The copyright holder for this preprint (which was not certified by peer review) is the author/funder, who has granted bioRxiv a license to display the preprint in perpetuity. It is made available under aCC-BY 4.0 International license.

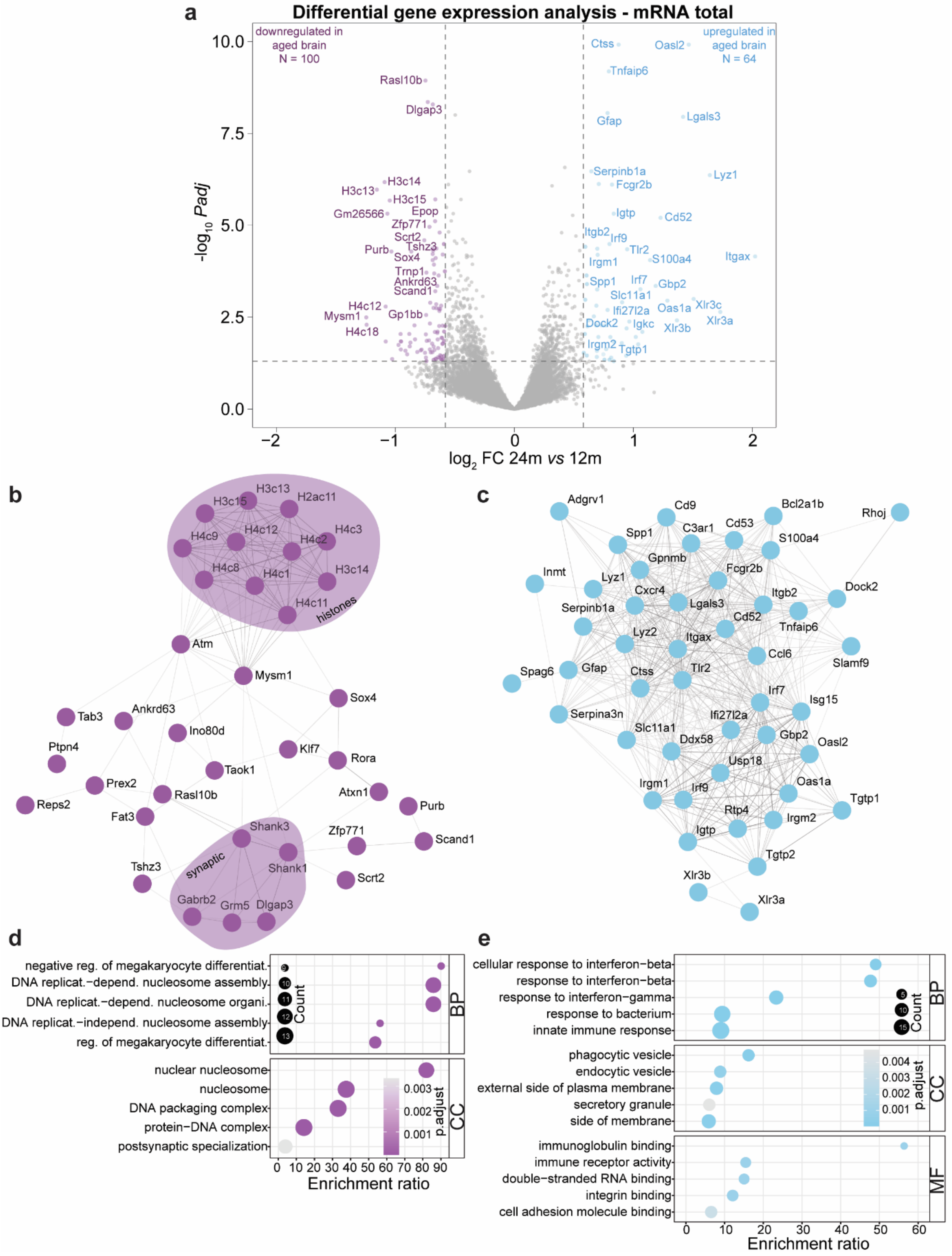

Extended Data Figure 4: Gene expression changes in the aging mouse brain for 24m vs 12m in mRNA-total fraction. (**a**) Volcano plot displaying the differentially expressed genes for 24m vs 12m. Inset: dot plot for biological replicate variability calculated using PCA, where sky blue represents increased expression with aging and medium orchid denotes decreased expression with aging. (**b, c**) STRING representation for the top 50 most (**b**) down- or (**c**)upregulated genes in 24m brain (respectively medium orchid and light blue). (**d, e**) GO-ORA of genes that are significantly down- or upregulated in 24m (*padj* ≤ 0.05, $|\log_2 FC| \geq 0.58$.

bioRxiv preprint doi: https://doi.org/10.1101/2025.08.14.669896; this version posted August 19, 2025. The copyright holder for this preprint (which was not certified by peer review) is the author/funder, who has granted bioRxiv a license to display the preprint in perpetuity. It is made available under aCC-BY 4.0 International license.

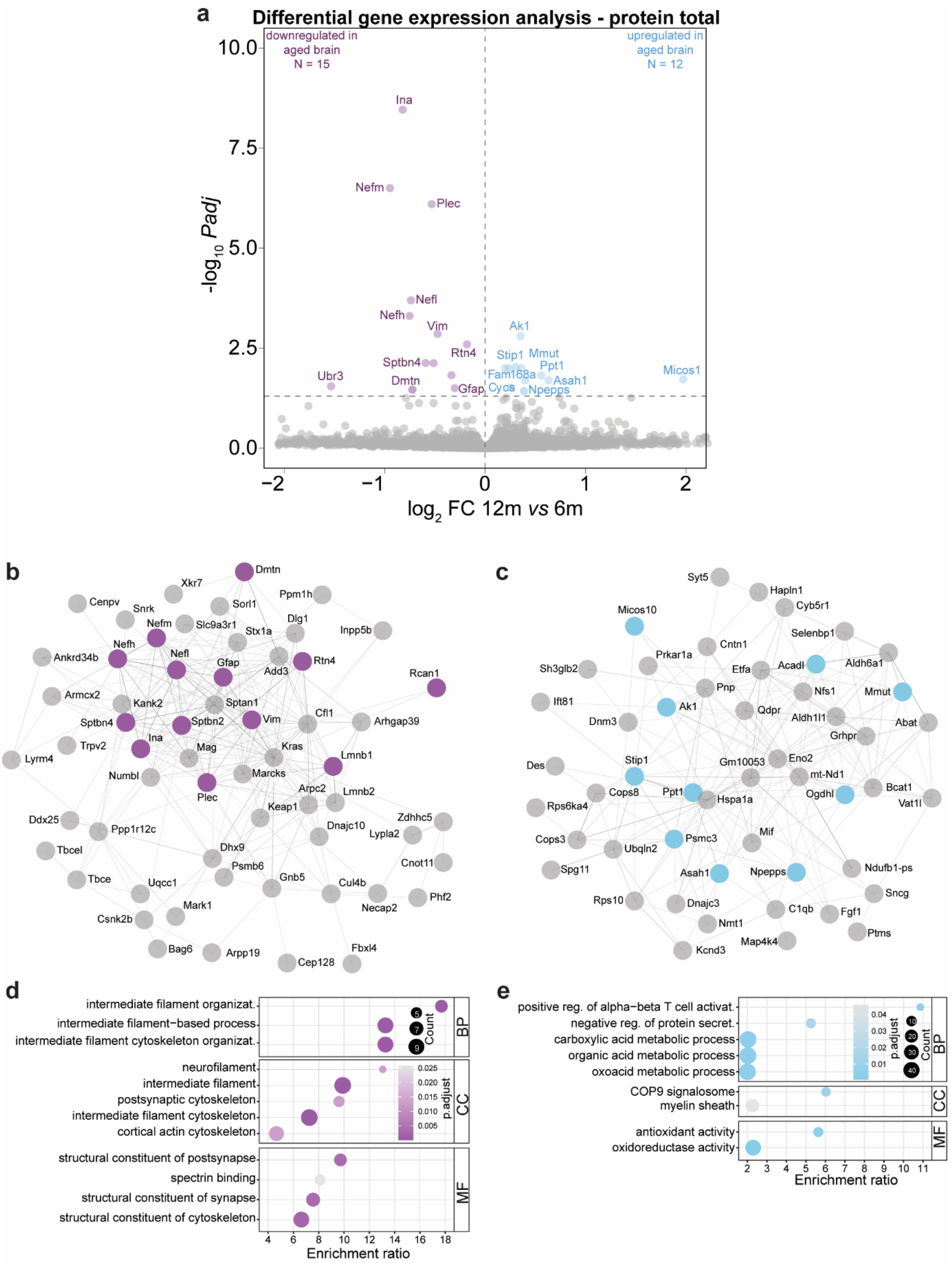

Extended Data Figure 5: Protein expression changes in the aging mouse brain for 12m vs 6m in protein-total fraction. (**a**) Volcano plot displaying the differentially expressed proteins for 12m vs 6m. Inset: dot plot for biological replicate variability calculated using PCA, where sky blue represents increased expression with aging and medium orchid denotes decreased expression with aging. (**b, c**) STRING representation for the top 50 most (**b**) down- or (**c**)upregulated proteins in 12m brain (respectively medium orchid and light blue), grey are the proteins that are not significant. (**d, e**) GO-ORA of the top150 proteins that are down- or upregulated in 12m**.**

bioRxiv preprint doi: https://doi.org/10.1101/2025.08.14.669896; this version posted August 19, 2025. The copyright holder for this preprint (which was not certified by peer review) is the author/funder, who has granted bioRxiv a license to display the preprint in perpetuity. It is made available under aCC-BY 4.0 International license.

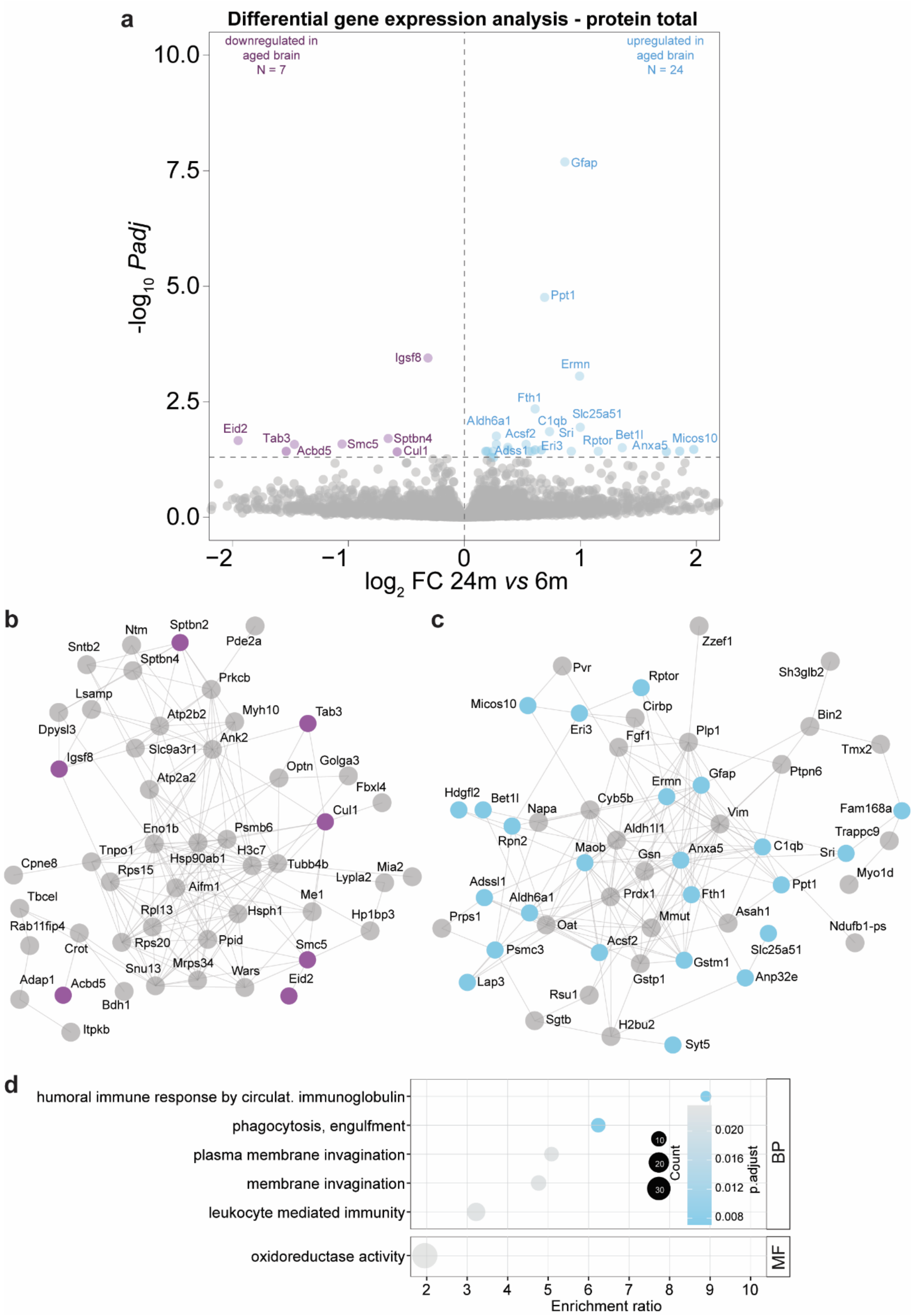

Extended Data Figure 6: Protein expression changes in the aging mouse brain for 24m vs 6m in protein-total fraction. (**a**) Volcano plot displaying the differentially expressed proteins for 24m vs 6m. Inset: dot plot for biological replicate variability calculated using PCA, where sky blue represents increased expression with aging and medium orchid denotes decreased expression with aging. (**b, c**) STRING representation for the top 50 most (**b**) down- or (**c**)upregulated proteins in 24m brain (respectively medium orchid and light blue), grey are the proteins that are not significant. (**d, e**) GO-ORA of the top150 proteins that are down- or upregulated in 24m**.**

bioRxiv preprint doi: https://doi.org/10.1101/2025.08.14.669896; this version posted August 19, 2025. The copyright holder for this preprint (which was not certified by peer review) is the author/funder, who has granted bioRxiv a license to display the preprint in perpetuity. It is made available under aCC-BY 4.0 International license.

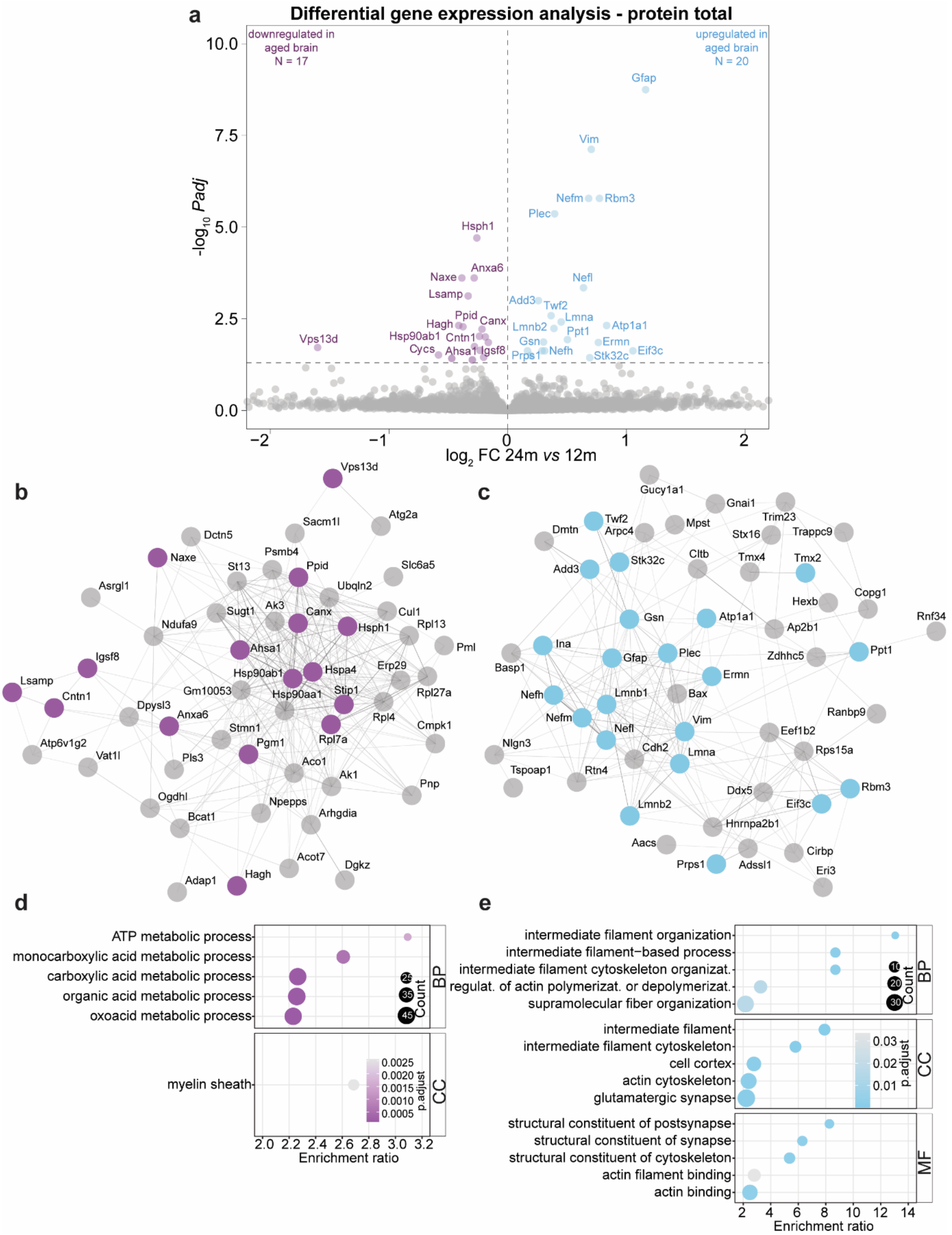

Extended Data Figure 7: Protein expression changes in the aging mouse brain for 24m vs 12m in protein-total fraction. (**a**) Volcano plot displaying the differentially expressed proteins for 24m vs 12m. Inset: dot plot for biological replicate variability calculated using PCA, where sky blue represents increased expression with aging and medium orchid denotes decreased expression with aging. (**b, c**) STRING representation for the top 50 most (**b**) down- or (**c**)upregulated proteins in 24m brain (respectively medium orchid and light blue), grey are the proteins that are not significant. (**d, e**) GO-ORA of the top150 proteins that are down- or upregulated in 24m.

bioRxiv preprint doi: https://doi.org/10.1101/2025.08.14.669896; this version posted August 19, 2025. The copyright holder for this preprint (which was not certified by peer review) is the author/funder, who has granted bioRxiv a license to display the preprint in perpetuity. It is made available under aCC-BY 4.0 International license.

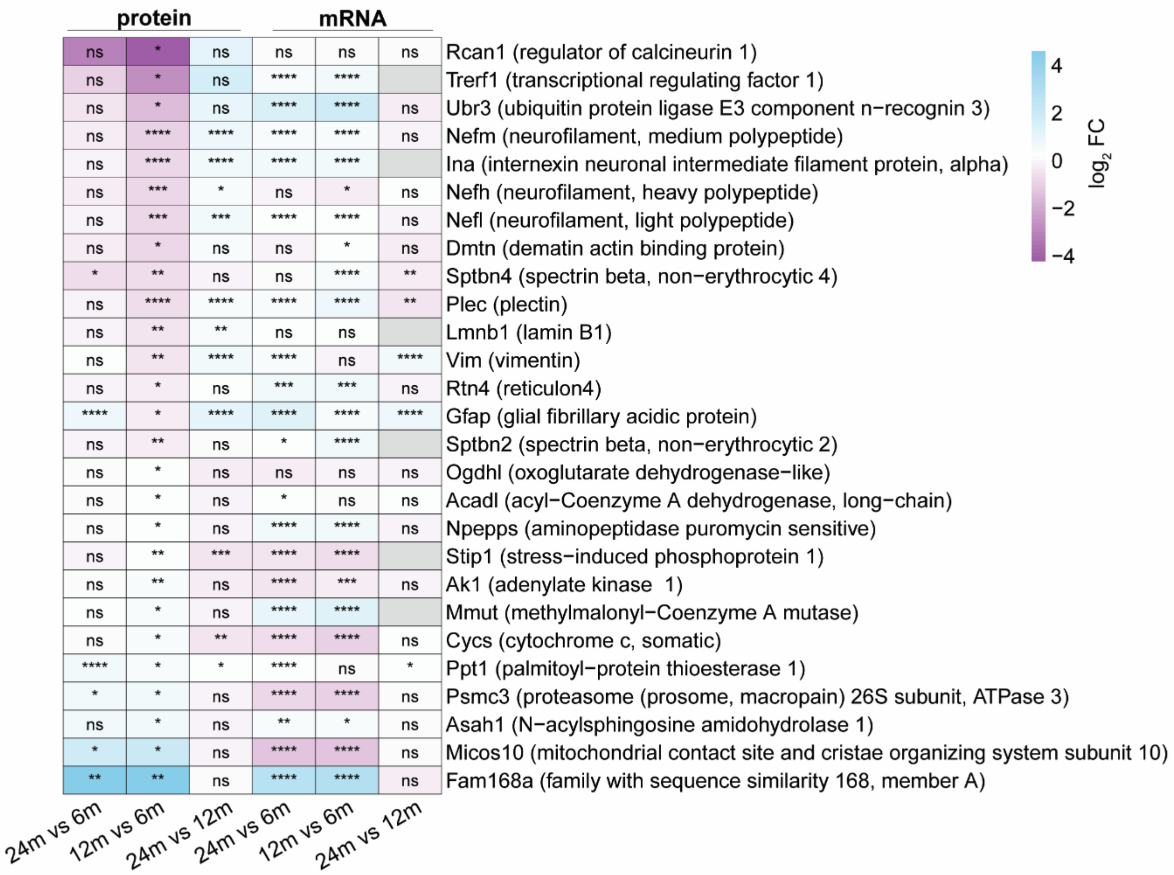

Extended Data Figure 8: Heatmap of proteins significantly altered in the 12m vs. 6m comparison for the protein- and mRNA -total Fraction. The heatmap displays proteins that show significant changes between 12 months and 6 months in the protein-total fraction. Sky blue indicates proteins with increased expression during aging, while medium orchid denotes proteins with decreased expression during aging.

bioRxiv preprint doi: https://doi.org/10.1101/2025.08.14.669896; this version posted August 19, 2025. The copyright holder for this preprint (which was not certified by peer review) is the author/funder, who has granted bioRxiv a license to display the preprint in perpetuity. It is made available under aCC-BY 4.0 International license.

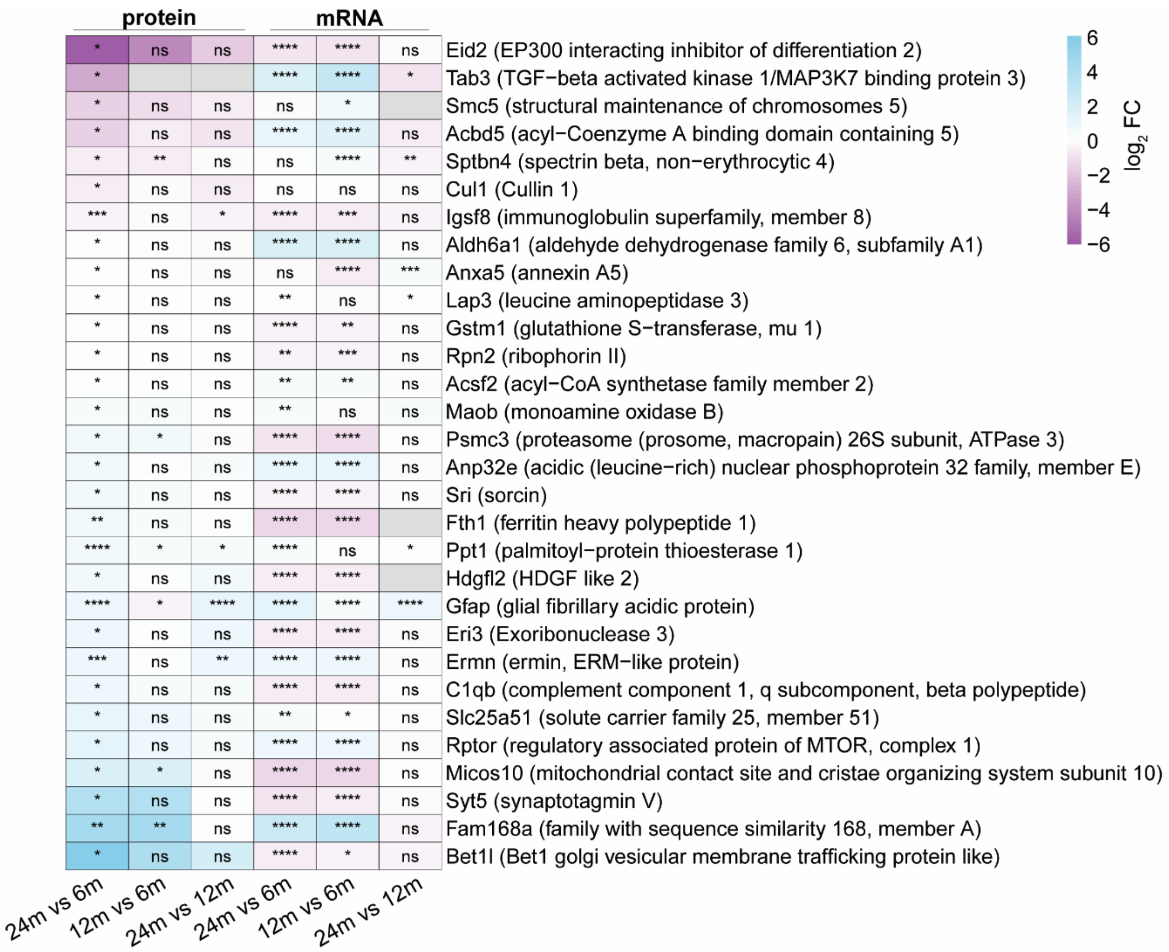

1496 **Extended Data Figure 9: Heatmap of proteins significantly altered in the 24m vs. 6m comparison for the**
1497 **protein- and mRNA -total Fraction.** The heatmap displays proteins that show significant changes between 24
1498 months and 6 months in the protein-total fraction. Sky blue indicates proteins with increased expression during
1499 aging, while medium orchid denotes proteins with decreased expression during aging.
1500

bioRxiv preprint doi: https://doi.org/10.1101/2025.08.14.669896; this version posted August 19, 2025. The copyright holder for this preprint (which was not certified by peer review) is the author/funder, who has granted bioRxiv a license to display the preprint in perpetuity. It is made available under aCC-BY 4.0 International license.

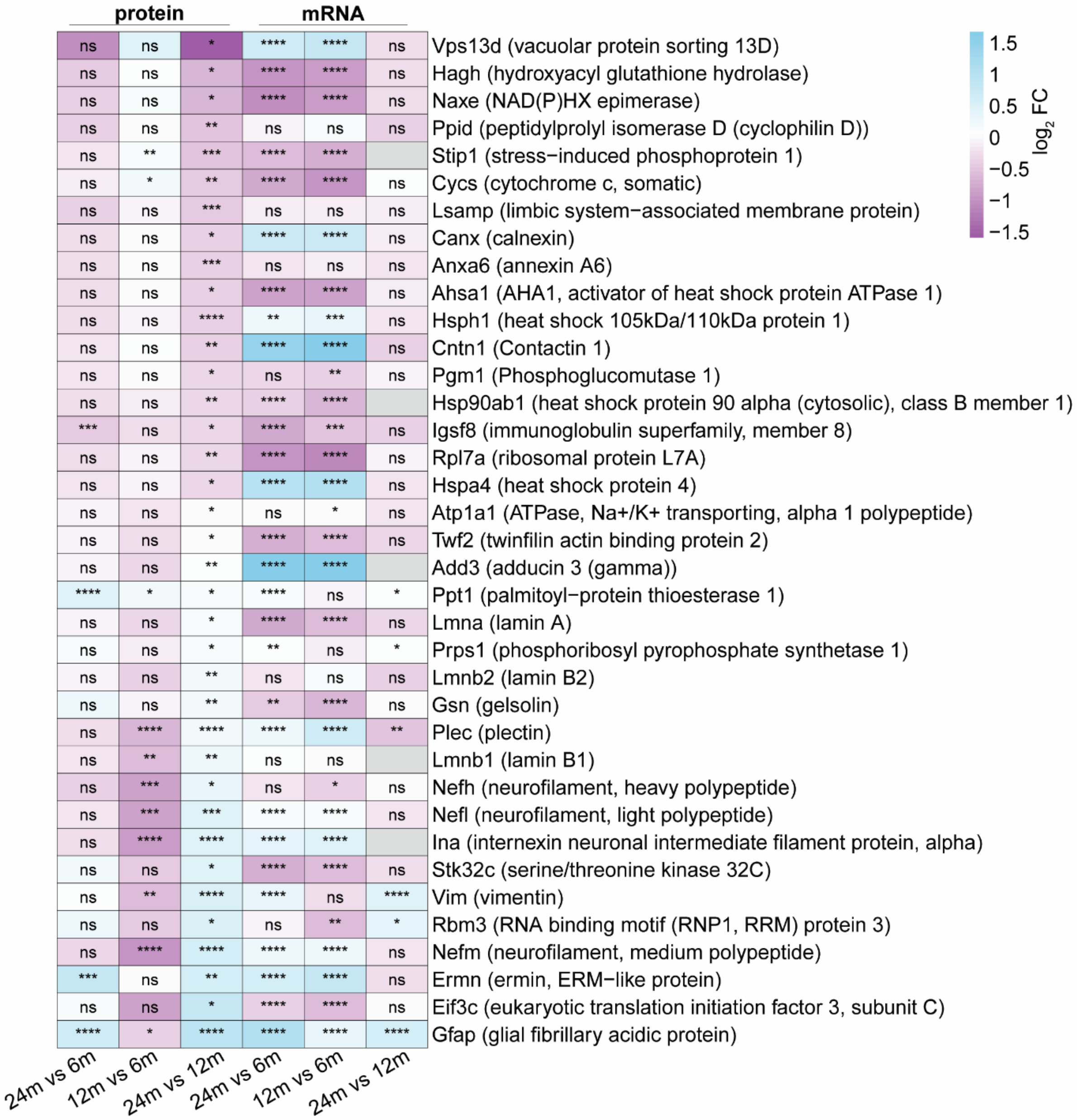

Extended Data Figure 10: Heatmap of proteins significantly altered in the 24m vs. 12m comparison for the protein- and mRNA -total Fraction. The heatmap displays proteins that show significant changes between 24 months and 12 months in the protein-total fraction. Sky blue indicates proteins with increased expression during aging, while medium orchid denotes proteins with decreased expression during aging.

bioRxiv preprint doi: https://doi.org/10.1101/2025.08.14.669896; this version posted August 19, 2025. The copyright holder for this preprint (which was not certified by peer review) is the author/funder, who has granted bioRxiv a license to display the preprint in perpetuity. It is made available under aCC-BY 4.0 International license.

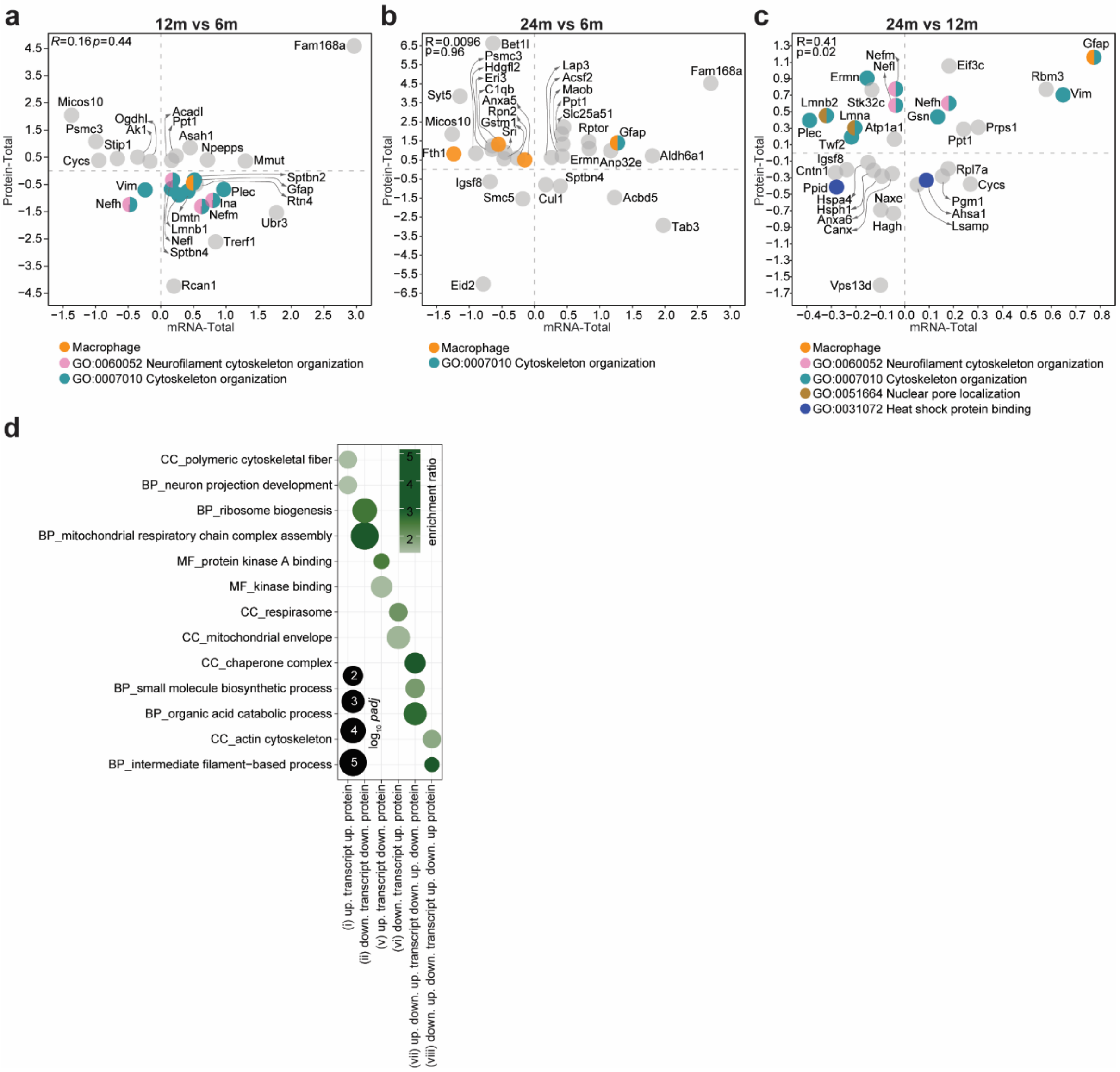

1506
1507 **Extended Data Figure 11: Comparison of the protein- and mRNA -total Fraction. (a-c)** Scatter plot between
1508 mRNA and protein for the proteins that show significant changes between (**a**) 12m and 6m (**b**) 24m and 6m (**c**)
1509 24m and 12m in the protein-total fraction. (**d**) GO-ORA of the non-linear dynamic patterns, here are top ~2 GO
1510 terms based on the enrichment ratio. For all patterns in detail refer to **Supplementary Table 1**. Dot sizes in the
1511 enrichment graphs correspond to the padj and color scales represents the enrichment ratio for each GO term, dark
1512 green – highly enriched and gray – less enriched.

bioRxiv preprint doi: https://doi.org/10.1101/2025.08.14.669896; this version posted August 19, 2025. The copyright holder for this preprint (which was not certified by peer review) is the author/funder, who has granted bioRxiv a license to display the preprint in perpetuity. It is made available under aCC-BY 4.0 International license.

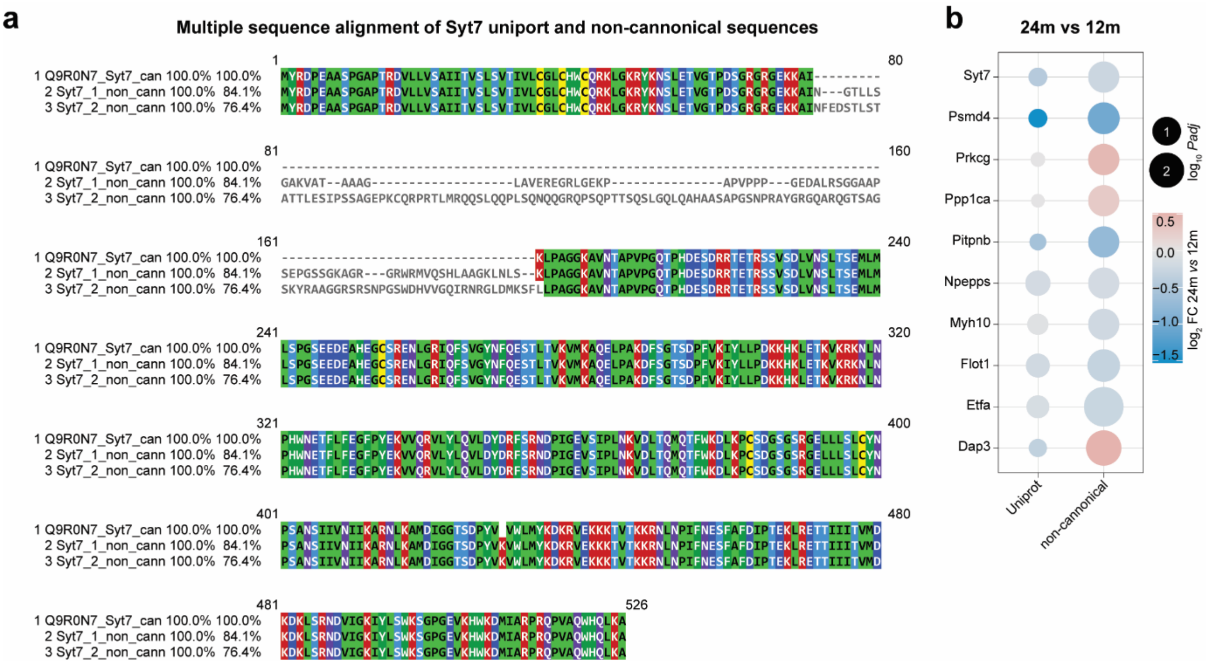

Extended Data Figure 12: Integration of proteo-transcriptomic datasets leads to the identification of novel proteoforms with aging. (**a**) Multiple sequence alignment of Syt7 canonical (can. the uniport sequence, Q9R0N7) and 2 non-canonical (non_cann.) proteoforms (Syt7_1, Syt7_2). (**b**) Dot plot for proteoforms that are differentially expressed at 24m vs. 12m (here the Syt7_1 is shown for the non-canonical as this showed significant difference). Dot sizes correspond to the absolute $\log_{10}$padj and color scales represents the $\log_2$FC for protein, red – upregulated and blue – downregulated at 24m compared to 12m.

bioRxiv preprint doi: https://doi.org/10.1101/2025.08.14.669896; this version posted August 19, 2025. The copyright holder for this preprint (which was not certified by peer review) is the author/funder, who has granted bioRxiv a license to display the preprint in perpetuity. It is made available under aCC-BY 4.0 International license.

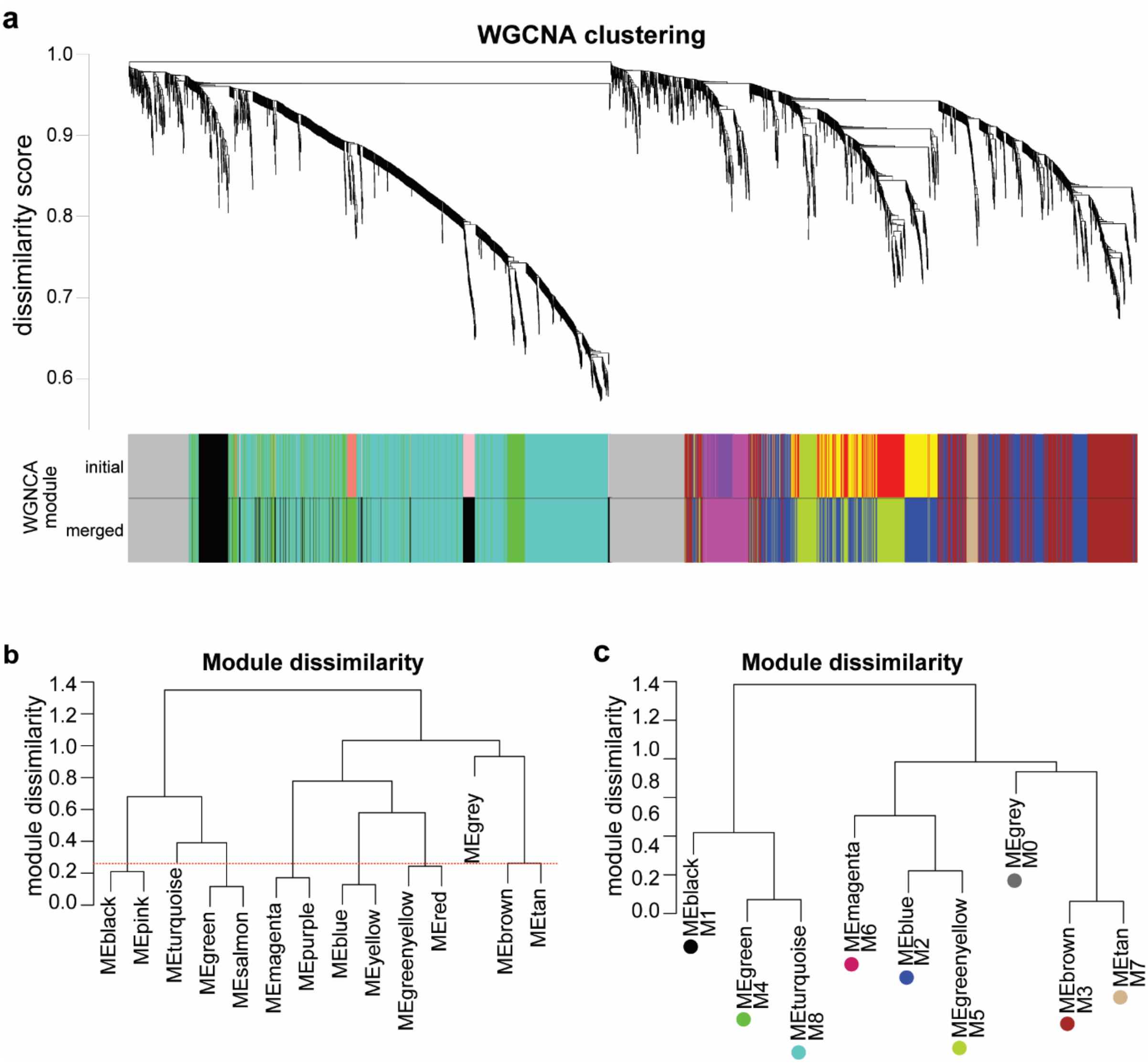

Extended Data Figure 13: Co-expression modules WO merging for p/m coefficient in the aging mouse brain. (**a**) WGNCA dendrogram with highlighted modules (lower colored bar). Genes were clustered based on a dissimilarity measure. The branches are modules of closely correlated gene groups that have a similar p/m coefficient. (**b**) Module dissimilarity based on module eigengene distances WO merge. Modules are grouped based on their p/m coefficient. (**c**) Module dissimilarity based on module eigengene distances after merging. Modules are grouped based on their p/m coefficient.

bioRxiv preprint doi: https://doi.org/10.1101/2025.08.14.669896; this version posted August 19, 2025. The copyright holder for this preprint (which was not certified by peer review) is the author/funder, who has granted bioRxiv a license to display the preprint in perpetuity. It is made available under aCC-BY 4.0 International license.

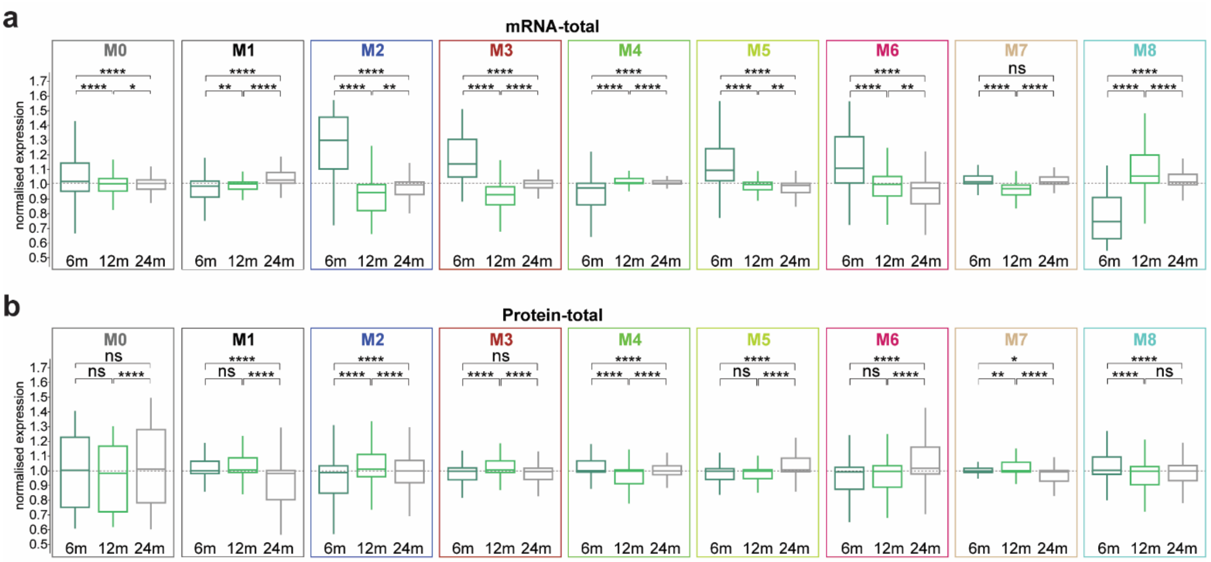

Extended Data Figure 14: mRNA and protein level changes in the co-expression modules across ages. **(a, b)** Boxplots of normalized (**a**) mRNA-total (**b**) protein-total in 6m, 12m and 24m mouse brain, grouped by their modules detected in the WGNCA method. Tukey posthoc test P-value * ≤ 0.05, ** ≤ 0.01, *** ≤ 0.001 and **** ≤ 0.0001.

bioRxiv preprint doi: https://doi.org/10.1101/2025.08.14.669896; this version posted August 19, 2025. The copyright holder for this preprint (which was not certified by peer review) is the author/funder, who has granted bioRxiv a license to display the preprint in perpetuity. It is made available under aCC-BY 4.0 International license.

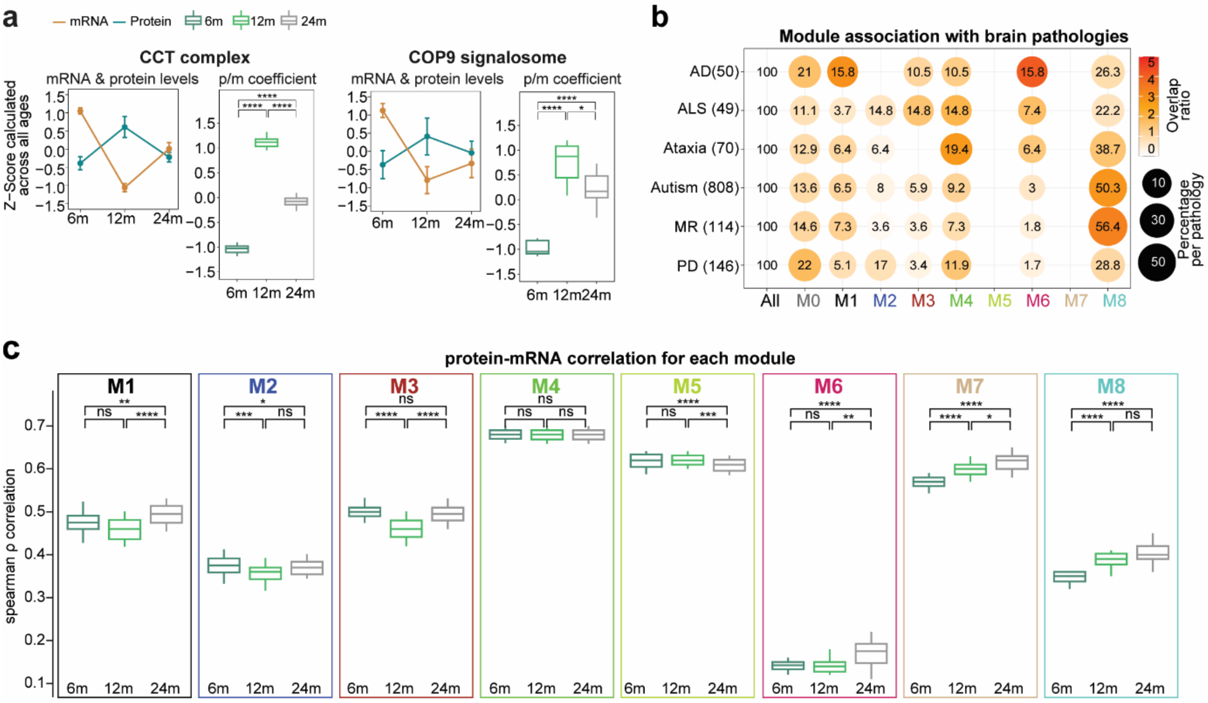

Extended Data Figure 15: mRNA, protein and p-m coefficient change in the aging brain and its association to brain pathologies. (**a**) Line plots for mRNA and protein trajectories and boxplot for p/m coefficient for mitochondrial complexes. Line colors reflect R color codes, with "peru" representing mRNA and "turquoise4" representing protein. P-values indicate the results of paired t-test followed by Tukey posthoc test P-value * ≤ 0.05, ** ≤ 0.01, *** ≤ 0.001 and **** ≤ 0.0001. (**b**) Dot plot for brain pathologies. Dot sizes correspond to the percentage of genes per pathology and color scales represents the overlap ratio for each pathology, dark orange (higher overlap) and white (lower overlap). (**c**) Boxplots of spearman ρ correlation for each age group between protein and mRNA within each module. P-values indicate the results of paired t-test followed by Tukey posthoc test P-value * ≤ 0.05, ** ≤ 0.01, *** ≤ 0.001 and **** ≤ 0.0001.

bioRxiv preprint doi: https://doi.org/10.1101/2025.08.14.669896; this version posted August 19, 2025. The copyright holder for this preprint (which was not certified by peer review) is the author/funder, who has granted bioRxiv a license to display the preprint in perpetuity. It is made available under aCC-BY 4.0 International license.

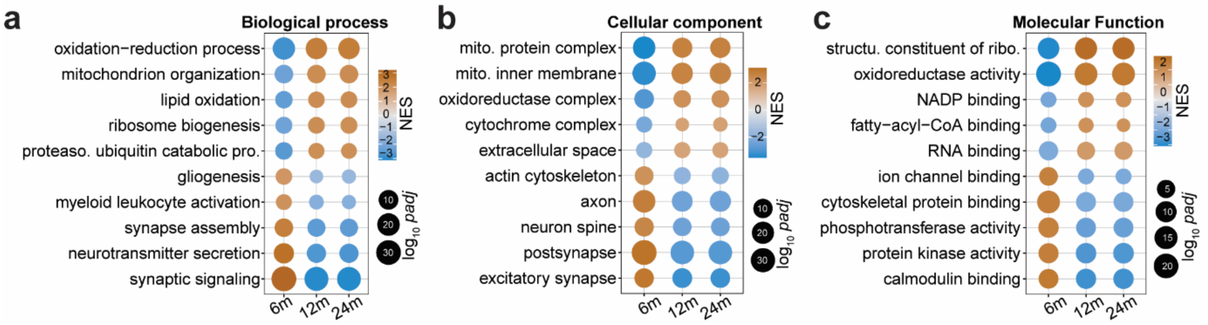

Extended Data Figure 16: p/m coefficient changes in each age group. (a-c) Gene ontology (GO)- Gene set enrichment analysis (GSEA) of genes that have an increased or decreased p/m coefficient for each age. Positive coefficient values (goldenrod) indicate increased protein levels relative to mRNA, suggesting efficient translation; negative values (skyblue) indicate reduced protein levels relative to mRNA, indicating inefficient translation. The size of the dots corresponds to $\log_{10}$*padj*, bigger the size of the bubble means they are highly significant (**a**) Biological process, (**b**) Cellular component and (**c**) Molecular function.

bioRxiv preprint doi: https://doi.org/10.1101/2025.08.14.669896; this version posted August 19, 2025. The copyright holder for this preprint (which was not certified by peer review) is the author/funder, who has granted bioRxiv a license to display the preprint in perpetuity. It is made available under aCC-BY 4.0 International license.

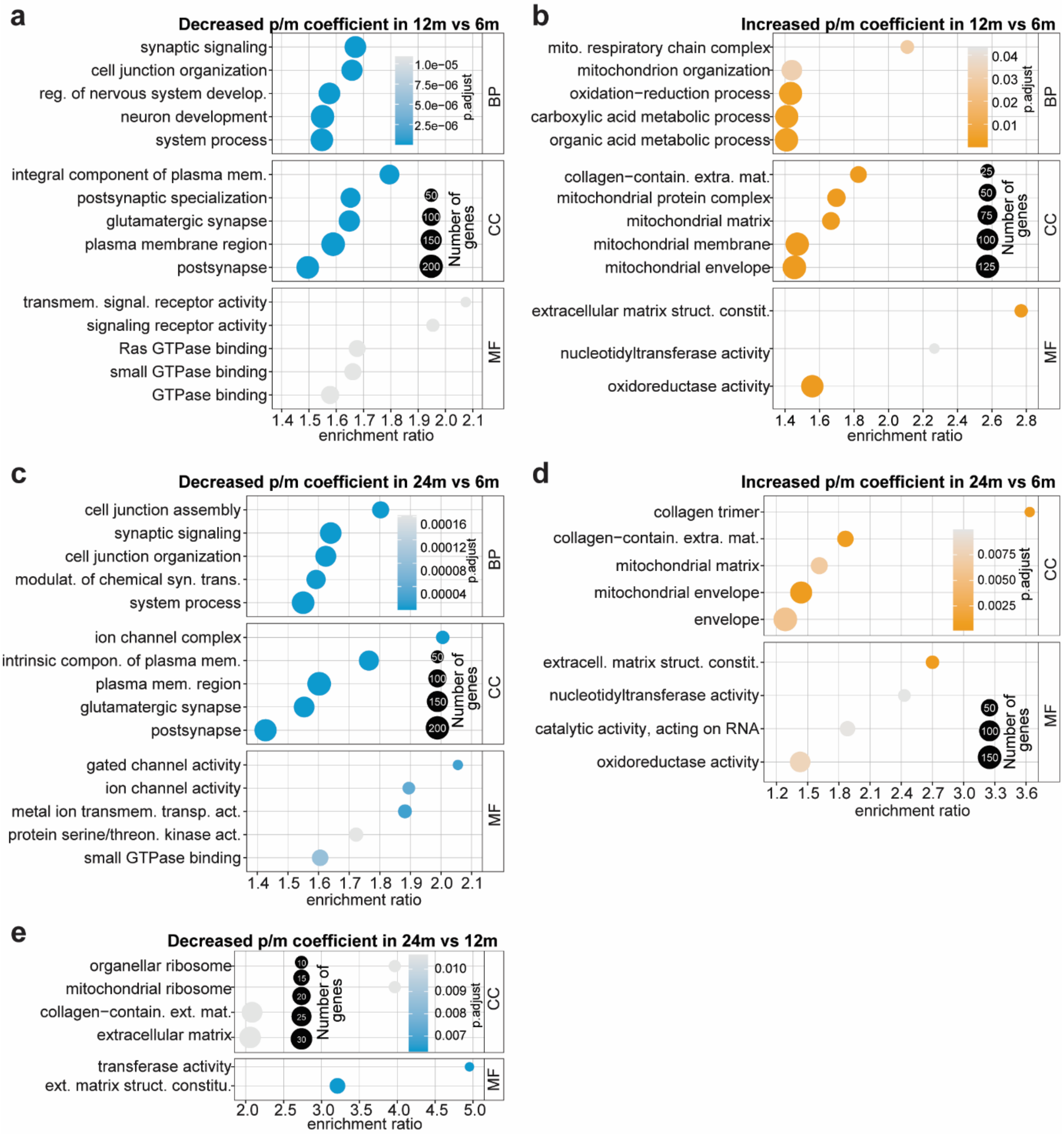

Extended Data Figure 17: GO-ORA of genes that have an increased or decreased p/m coefficient for each age comparison. (**a**) Decreased p/m coefficient 12m vs 6m (**b**) Increased p/m coefficient 12m vs 6m (**c**) Decreased p/m coefficient 24m vs 6m (**d**) Increased p/m coefficient 24m vs 6m (**e**) Decreased p/m coefficient 24m vs 12m. Colour scale corresponds to $\log_{10}$*padj*, Goldenrod color indicate increased p/m coefficient (protein levels increased relative to mRNA), suggesting efficient translation; Skyblue color indicate decreased p/m coefficient (reduced protein levels relative to mRNA), indicating inefficient translation. Gray is less significant. The size of the dots corresponds to the number of genes, bigger the size of the bubble means they are highly significant.

bioRxiv preprint doi: https://doi.org/10.1101/2025.08.14.669896; this version posted August 19, 2025. The copyright holder for this preprint (which was not certified by peer review) is the author/funder, who has granted bioRxiv a license to display the preprint in perpetuity. It is made available under aCC-BY 4.0 International license.

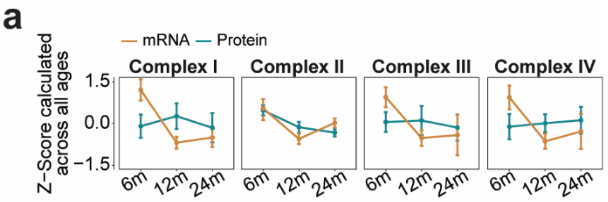

Extended Data Figure 18: mRNA and protein levels in mitochondrial complexes (**a**) Line plots for mRNA and protein trajectories for mitochondrial complexes. Line colors reflect R color codes, with "peru" representing mRNA and "turquoise4" representing protein.

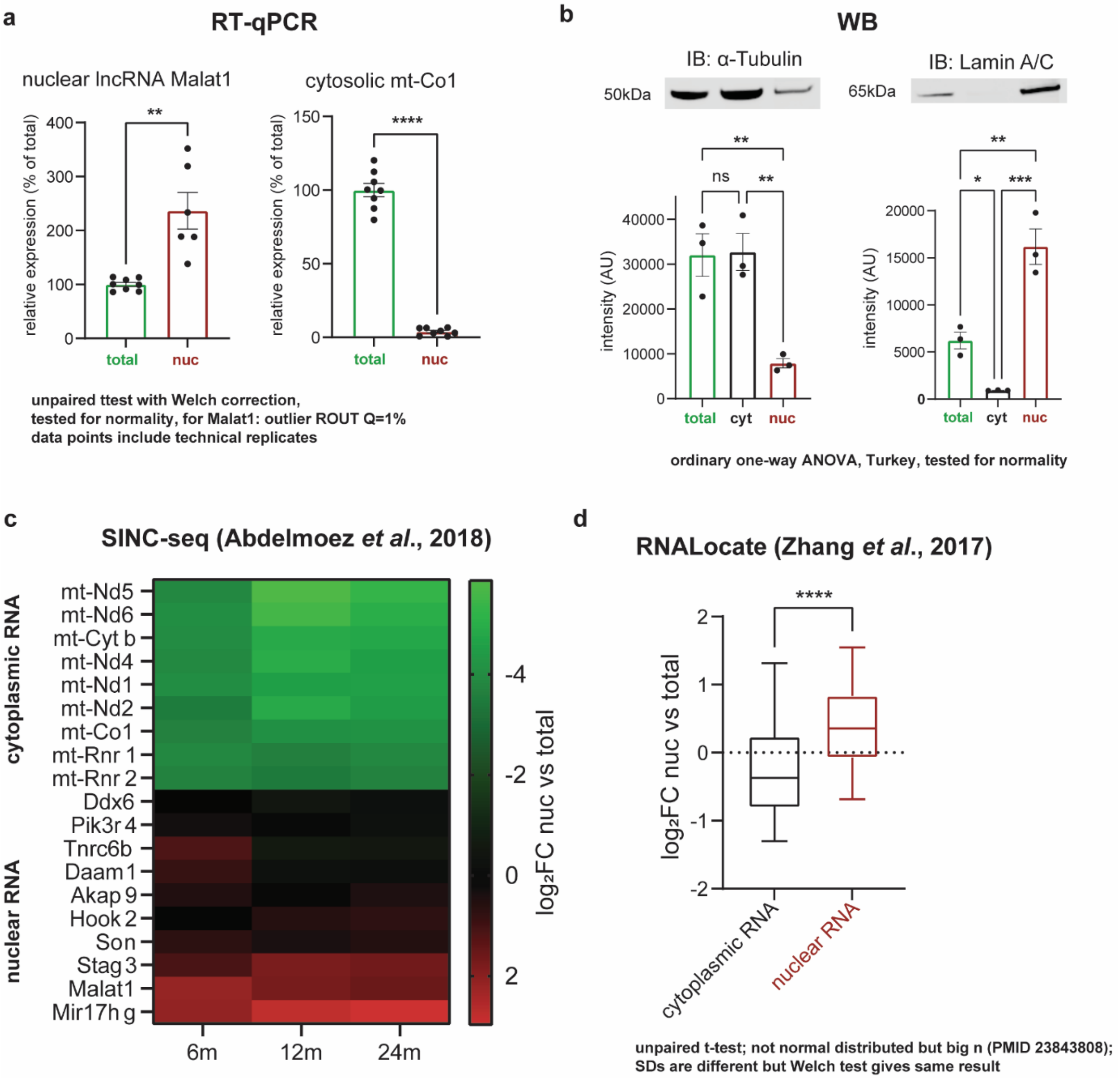

Extended Data Figure 19*:* Quality control of subcellular fractionation for RNA-Isolation and subsequent sequencing. (**a**) Reverse-transcription qualitative PCR (RT-qPCR) confirms enrichment of nuclear transcript *Malat1* in the nuclear fraction (nuc). Reversely, cytosolic *mt-Co1* is strongly depleted in the nuclear fraction. Unpaired t-test with Welch correction; n=6 technical replicates from 3 biological samples. (**b)** Western blot analysis of cytosolic protein α-Tubulin, which is depleted in the nuclear fraction (nuc), and nuclear lamin Lamin A/C, which is depleted in the cytosolic fraction (Cyt) and relatively enriched in Nuc. Ordinary one-way ANOVA with Turkey correction; n=3 biological replicates. (**c)** Comparison of nuclear vs total enrichment (log_2FC Nuc vs Total) of our data with single-cell integrated nuclear RNA and cytoplasmic RNA sequencing (SINC-seq[53]) showing that transcripts identified by SINC-seq as nuclear are indeed enriched in the nucleus in my dataset and cytoplasmic transcripts are de-enriched. (**d)** Comparison with annotations from RNALocate, a web-accessible database providing subcellular RNA localization[54]. Fold changes of nuclear enrichment (log_2FC Nuc vs Total) are significantly higher, meaning nuclear enrichment, among transcripts that are annotated as 'nuclear RNA'. Unpaired t-test with Welch correction of 1465 and 2259 transcripts with cytoplasmic and nuclear annotation, respectively. Boxplots show median, 25th to 75th percentile as box and 5th to 95th percentile as whiskers. * *p*<0.5, ** *p*<0.01, *** *p*<0.001, **** *p*<0.0001.

bioRxiv preprint doi: https://doi.org/10.1101/2025.08.14.669896; this version posted August 19, 2025. The copyright holder for this preprint (which was not certified by peer review) is the author/funder, who has granted bioRxiv a license to display the preprint in perpetuity. It is made available under aCC-BY 4.0 International license.

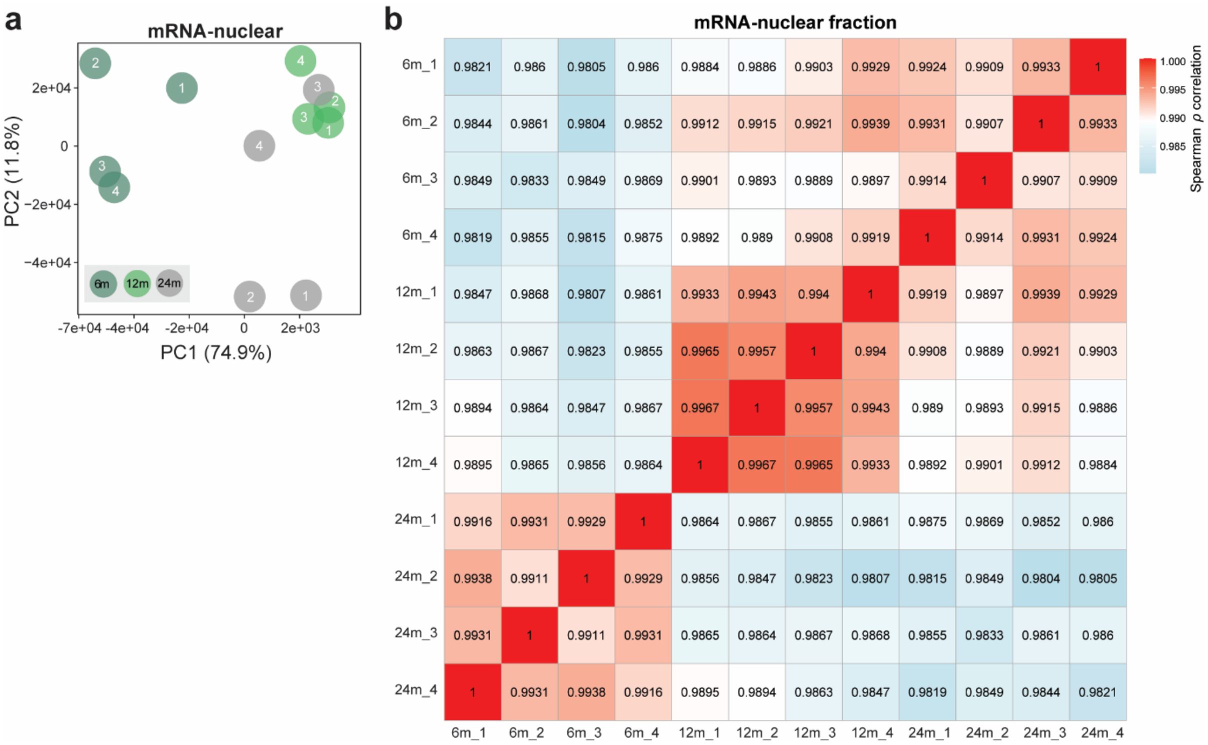

Extended Data Figure 20: nuclear-mRNA fraction correlation and principal component analysis. (a) Dot plots depict biological replicate variability via principal component analysis (PCA) for the mRNA – nuclear dataset. **(b)** Heatmap for the mRNA-nuclear fraction, with red indicating high correlation and sky blue indicating low correlation.

bioRxiv preprint doi: https://doi.org/10.1101/2025.08.14.669896; this version posted August 19, 2025. The copyright holder for this preprint (which was not certified by peer review) is the author/funder, who has granted bioRxiv a license to display the preprint in perpetuity. It is made available under aCC-BY 4.0 International license.

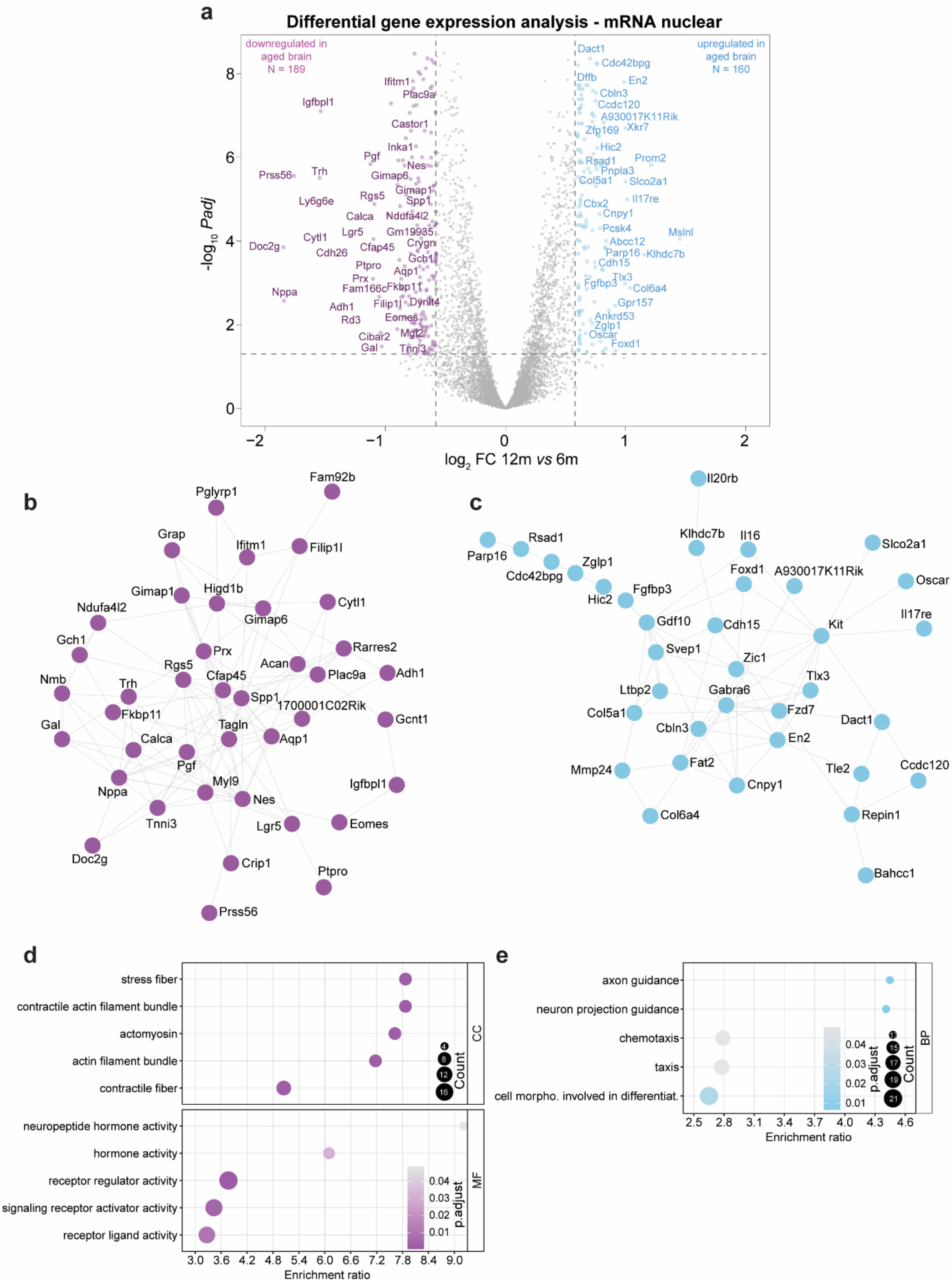

Extended Data Figure 21: Gene expression changes in the aging mouse brain for 12m vs 6m in mRNA-nuclear fraction. (**a**) Volcano plot displaying the differentially expressed genes for 12m vs 6m. Inset: dot plot for biological replicate variability calculated using PCA, where sky blue represents increased expression with aging and medium orchid denotes decreased expression with aging. (**b, c**) STRING representation for the top 50 most (**b**) down- or (**c**)upregulated genes in 12m brain (respectively medium orchid and light blue). (**d, e**) GO-ORA of genes that are significantly down- or upregulated in 12m (*padj* ≤ 0.05, $|log_2FC| \geq 0.58$**.**

bioRxiv preprint doi: https://doi.org/10.1101/2025.08.14.669896; this version posted August 19, 2025. The copyright holder for this preprint (which was not certified by peer review) is the author/funder, who has granted bioRxiv a license to display the preprint in perpetuity. It is made available under aCC-BY 4.0 International license.

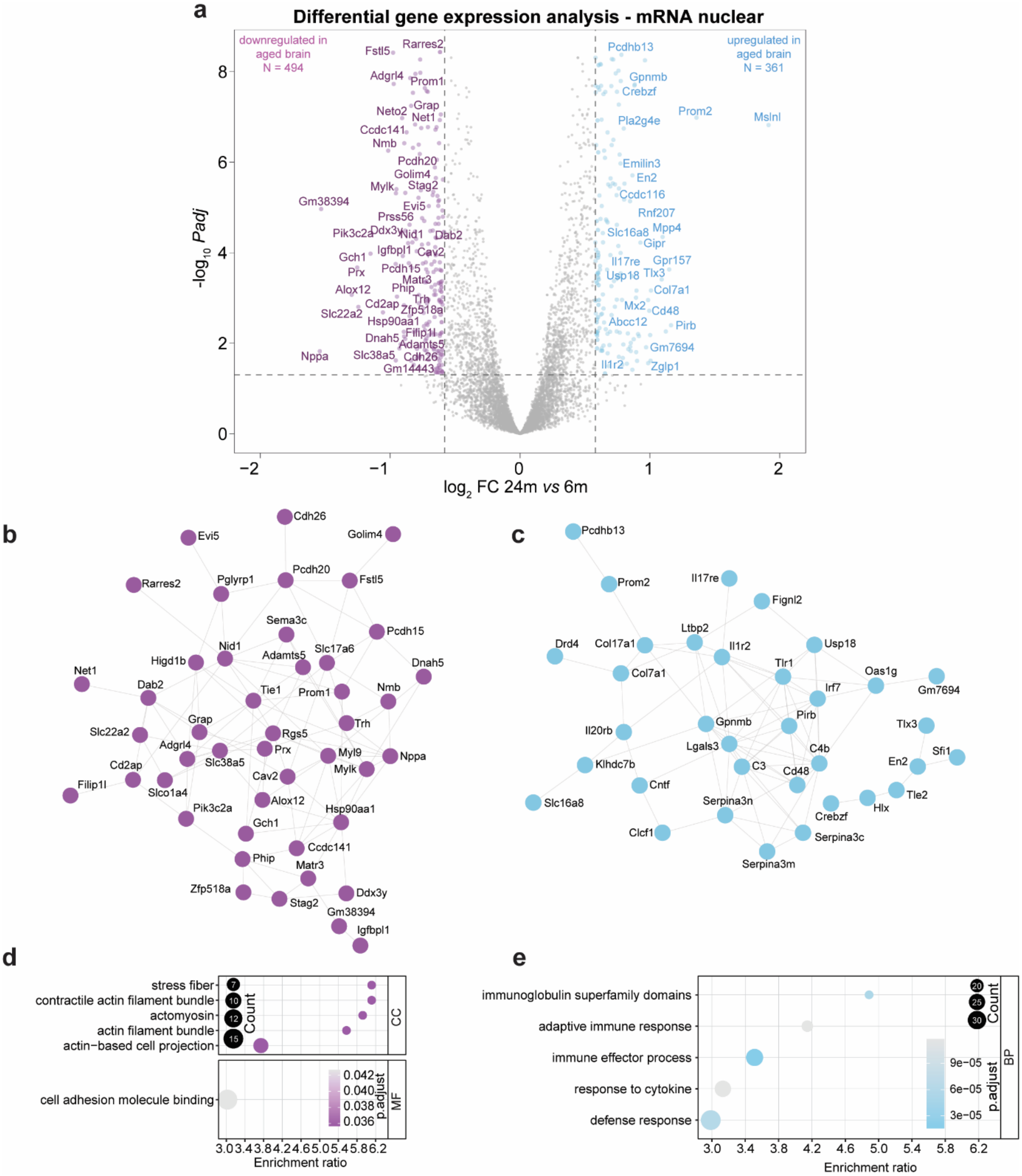

Extended Data Figure 22: Gene expression changes in the aging mouse brain for 24m vs 6m in mRNA-nuclear fraction. (**a**) Volcano plot displaying the differentially expressed genes for 24m vs 6m. Inset: dot plot for biological replicate variability calculated using PCA, where sky blue represents increased expression with aging and medium orchid denotes decreased expression with aging. (**b, c**) STRING representation for the top 50 most (**b**) down- or (**c**) upregulated genes in 24m brain (respectively medium orchid and light blue). (**d, e**) GO-ORA of genes that are significantly down- or upregulated in 24m (*padj* ≤ 0.05, $|\log_2 FC| \geq 0.58$**.**

bioRxiv preprint doi: https://doi.org/10.1101/2025.08.14.669896; this version posted August 19, 2025. The copyright holder for this preprint (which was not certified by peer review) is the author/funder, who has granted bioRxiv a license to display the preprint in perpetuity. It is made available under aCC-BY 4.0 International license.

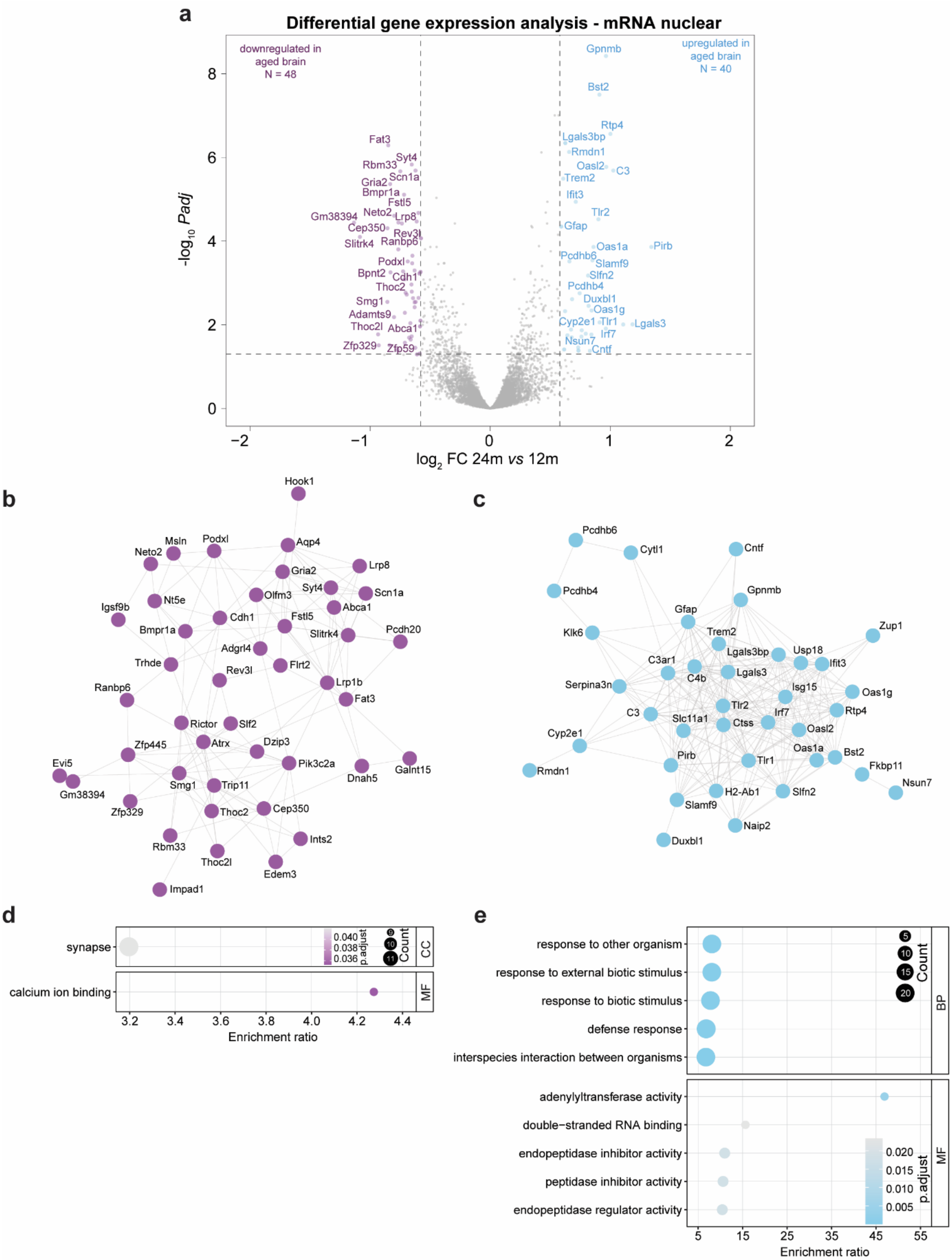

Extended Data Figure 23: Gene expression changes in the aging mouse brain for 24m vs 12m in mRNA-nuclear fraction. (**a**) Volcano plot displaying the differentially expressed genes for 24m vs 6m. Inset: dot plot for biological replicate variability calculated using PCA, where sky blue represents increased expression with aging and medium orchid denotes decreased expression with aging. (**b, c**) STRING representation for the top 50 most (**b**) down- or (**c**)upregulated genes in 24m brain (respectively medium orchid and light blue). (**d, e**) GO-ORA of genes that are significantly down- or upregulated in 24m (*padj* ≤ 0.05, |log$_2$FC| ≥ 0.58.

bioRxiv preprint doi: https://doi.org/10.1101/2025.08.14.669896; this version posted August 19, 2025. The copyright holder for this preprint (which was not certified by peer review) is the author/funder, who has granted bioRxiv a license to display the preprint in perpetuity. It is made available under aCC-BY 4.0 International license.

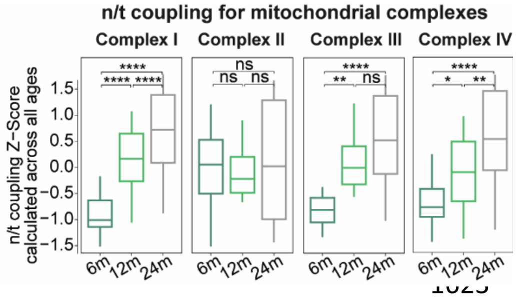

Extended Data Figure 24: n/t coupling for mitochondrial complexes. Boxplots for n/t coupling for mitochondrial complexes. P-values indicate the results of paired t-test followed by Tukey posthoc test P-value * ≤ 0.05, ** ≤ 0.01, *** ≤ 0.001 and **** ≤ 0.0001.

a p/m coefficient

	6m	12m	24m
Akt1	0.12	0.14	-0.26
Eif2a	-0.16	-0.15	0.31
Hspa5	1.11	-0.35	-0.76
Rptor	0.09	-0.13	0.05
Tsc1	1.01	-0.64	-0.37
Tsc2	0.19	0.22	-0.41

b mRNA-total

	6m	12m	24m
Akt1	0.29	0.20	-0.49
Eif2a	1.18	-0.67	-0.51
Hspa5	-1.13	0.33	0.80
Rptor	-1.31	0.76	0.55
Tsc1	-1.24	0.40	0.84
Tsc2	-0.32	0.67	-0.34

c protein-total

	6m	12m	24m
Akt1	0.14	0.15	-0.28
Eif2a	0.17	-0.31	0.14
Hspa5	-0.05	0.09	-0.05
Rptor	-0.43	0.17	0.27
Tsc1	0.49	-0.54	0.05
Tsc2	0.18	0.28	-0.45

(Color scale: Z-Score calculated across all ages; 1, 0, -1)

Extended Data Figure 25: Heatmap of mTOR and ER stress genes. (a) p/m coefficient (**b**) mRNA-total and (**c**) protein-total. Colors based on the color names in R indicate Z-scores across all ages, where red signifies increased abundance relative to all ages, and skyblue signifies decreased abundance.

bioRxiv preprint doi: https://doi.org/10.1101/2025.08.14.669896; this version posted August 19, 2025. The copyright holder for this preprint (which was not certified by peer review) is the author/funder, who has granted bioRxiv a license to display the preprint in perpetuity. It is made available under aCC-BY 4.0 International license.

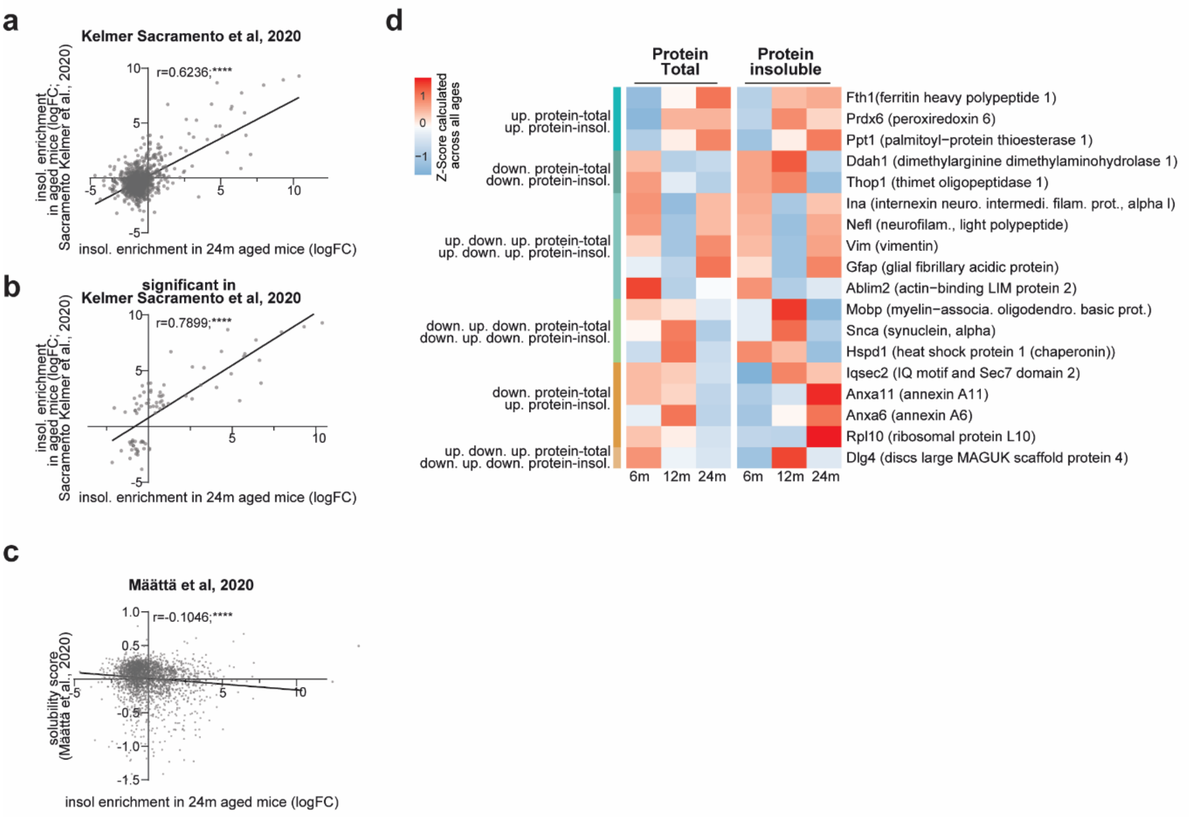

Extended Data Figure 26: Protein level changes in the aging mouse brain in protein-insoluble fraction. (a, b) Comparison of insoluble-enriched proteins between our study and the study by Kelmer Sacramento et al. (**a**) shows the correlation of all common proteins and their insoluble enrichment at 24 months between the two studies, (**b**) shows the correlation between the significantly enriched proteins from the Kelmer Sacramento study and our data. (**c**) Correlation of our insoluble enrichment data to the protein solubility score introduced by Määttä at al., 2020. Note that increasing negative solubility score indicates higher aggregation propensity. Correlations are indicated as Pearson correlation coefficient. * p<0.5, ** p<0.01, **** p<0.0001. (**d**) Heatmap illustrating six distinct patterns in protein total and insoluble levels across ages. Color gradient for the Z-scores across all ages (red increased and blue decreased).

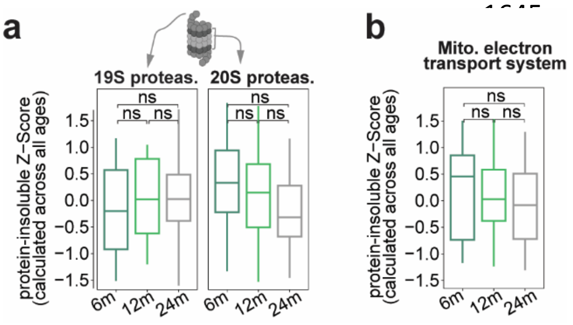

Extended Data Figure 27: Changes in the protein - insoluble levels for the proteasome and mitochondrial proteins. (a, b) Boxplots for protein aggregate for (**a**) 19S and 20S proteasome complexes, and (**b**) proteins of the mitochondrial electron transport system (ETS). P-values indicate the results of paired t-test followed by Tukey posthoc test P-value * ≤ 0.05, ** ≤ 0.01, *** ≤ 0.001 and **** ≤ 0.0001.

bioRxiv preprint doi: https://doi.org/10.1101/2025.08.14.669896; this version posted August 19, 2025. The copyright holder for this preprint (which was not certified by peer review) is the author/funder, who has granted bioRxiv a license to display the preprint in perpetuity. It is made available under aCC-BY 4.0 International license.

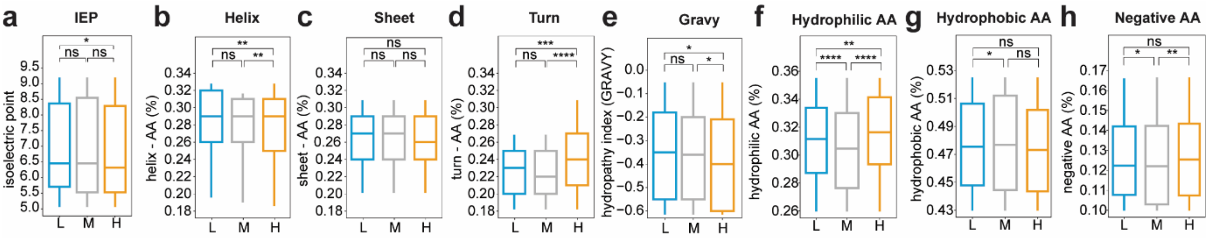

Extended Data Figure 28: Biochemical property alterations in the aged brain. (**a-h**) Boxplot for proteins for high (H, increased), low (L, decreased) and middle (M, unchanged) in 24m compared to 12m. (**a**) Isoelectric point (IEP), (**b**) Amino acid percentage for Helix, (**c**) Amino acid percentage for Sheet, (**d**) Amino acid percentage for Turn, (**e**) Amino acid percentage for Hydropathy index (Gravy), (**f**) Amino acid percentage for hydrophilic amino acids (**g**) Amino acid percentage for hydrophobic amino acids, and (**h**) Amino acid percentage for negative amino acids. Colors based on the color names in R indicate, 'goldenrod' indicate high (H), 'grey' indicate middle (M) and 'skyblue' indicate low (L). P-values indicate the results of paired t-test followed by Tukey posthoc test P-value * ≤ 0.05, ** ≤ 0.01, *** ≤ 0.001 and **** ≤ 0.0001.

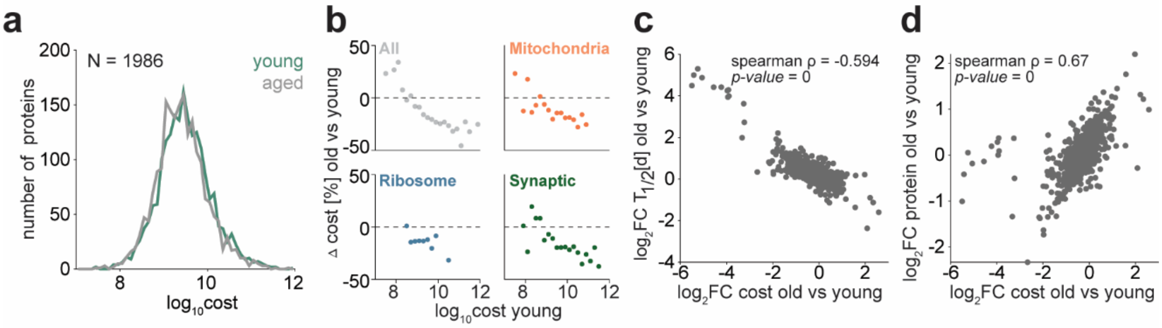

Extended Data Figure 29: Energy imbalance in the aged brain. (**a**) The distribution of cost per protein species spans four orders of magnitude in young and older mice, hence the global cost strongly depends on the most expensive proteins. Bin size is 0.1 (in log_{10} scale). (**b**) Changes of cost per protein species are negatively correlated with its energetic burden in younger mice. Proteins are binned (bin size 0.2 in log_{10} scale) based on their associated cost in younger age and with changes shown in percentage. Grey – all proteins, orange - mitochondrial proteins, blue - ribosomal and green - synaptic proteins. (**c,d**) Spearman ρ correlation for the change in cost per protein with age is negatively associated with its (**c**) half-life and positively with its (**d**) log_2FC.

bioRxiv preprint doi: https://doi.org/10.1101/2025.08.14.669896; this version posted August 19, 2025. The copyright holder for this preprint (which was not certified by peer review) is the author/funder, who has granted bioRxiv a license to display the preprint in perpetuity. It is made available under aCC-BY 4.0 International license.

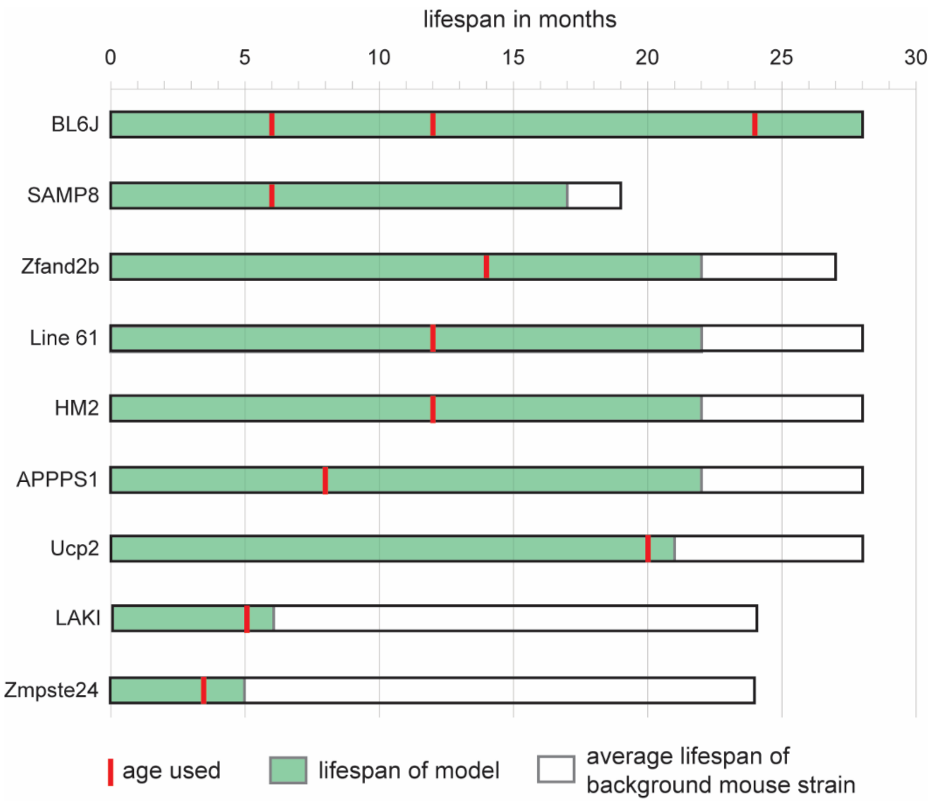

Extended Data Figure 30: Overview of average lifespan and used ages of mouse model cohorts used in this study. General average lifespan of the specific mouse strain is indicated in white. If the model has extended or decreased lifespan, this is indicated in the overlaid green. The red line marks the age used here. Age in months. Data on lifespan was retrieved from the original publications for each model (see Methods).

bioRxiv preprint doi: https://doi.org/10.1101/2025.08.14.669896; this version posted August 19, 2025. The copyright holder for this preprint (which was not certified by peer review) is the author/funder, who has granted bioRxiv a license to display the preprint in perpetuity. It is made available under aCC-BY 4.0 International license.

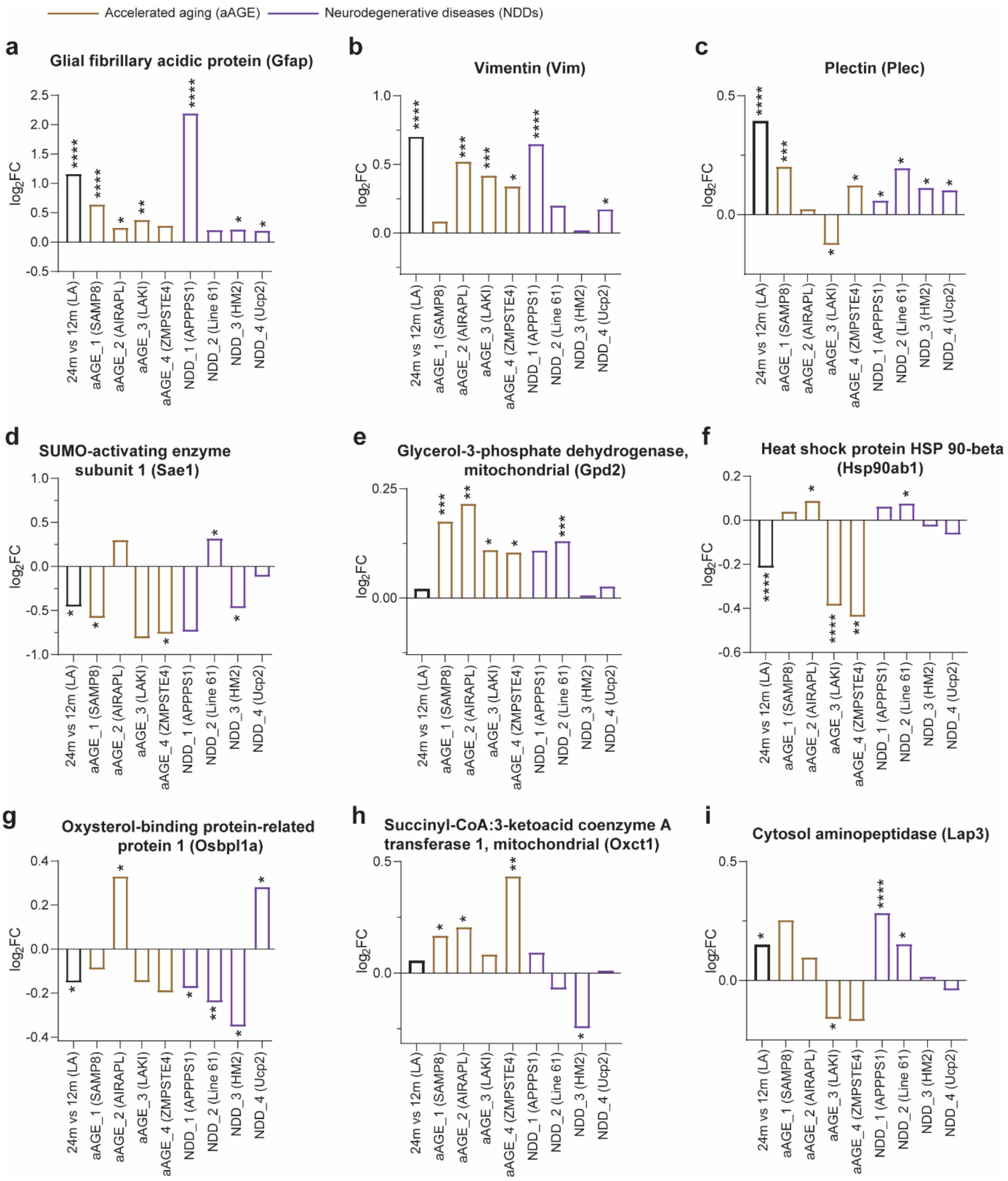

Extended Data Figure 31: Proteins found significantly changed in at least 5 mouse models, including physiological aging 24 vs 12. Bar plot for proteins that are significantly changed in atleast 5 mouse models and late aging (**a**) Glial fibrillary acidic protein (Gfap), (**b**) Vimentin (Vim), (**c**) Plectin (Plec), (**d**) SUMO1 activating enzyme subunit 1 (Sae1), (**e**) Glycerol-3-phosphate dehydrogenase (Gpd2), (**f**) Heat shock protein 90 alpha family class B member 1 (Hsp90ab1), (**g**) Oxysterol binding protein like 1A (Osbpl1a), (**h**) Succinyl-CoA:3-ketoacid coenzyme A transferase 1, mitochondrial, (**i**) Leucine aminopeptidase 3 (Lap3). 24m vs. 12m (LA) – black, accelerated aging (aAGE) in ‘peru’, neurodegenerative diseases (NDD) in purple. Stars indicate DEqMS significance in the respective comparison of 24 vs 12 or of model vs littermate control: * p<0.05, ** p<0.01, *** p<0.001, **** p<0.0001.

bioRxiv preprint doi: https://doi.org/10.1101/2025.08.14.669896; this version posted August 19, 2025. The copyright holder for this preprint (which was not certified by peer review) is the author/funder, who has granted bioRxiv a license to display the preprint in perpetuity. It is made available under aCC-BY 4.0 International license.

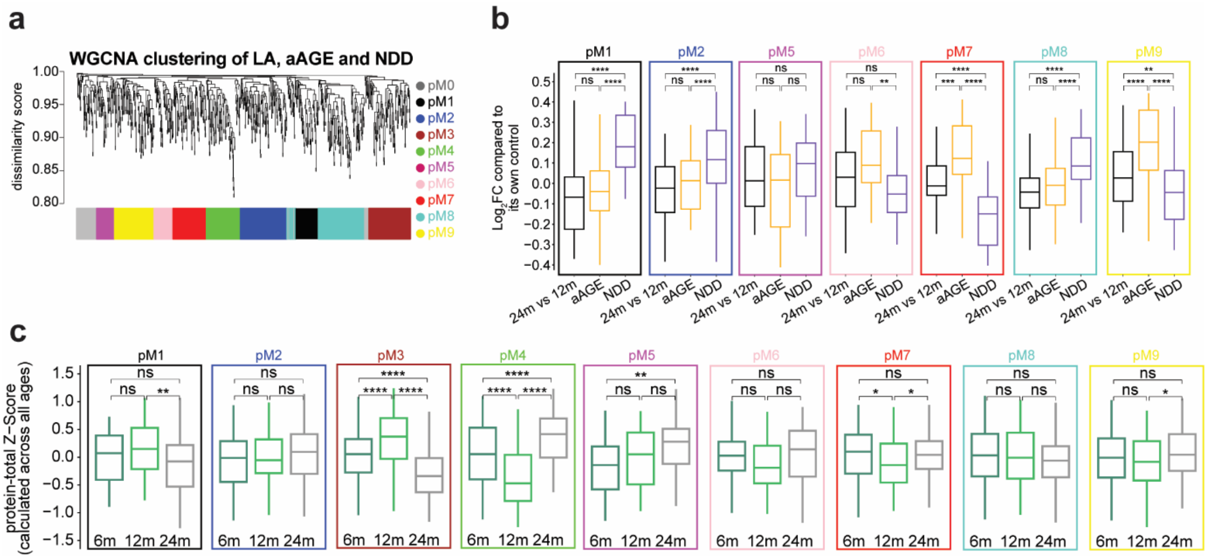

Extended Data Figure 32: Module level alterations in late aging, aAGE and NDD brain proteomes. (**a**) WGNCA dendrogram with highlighted modules (lower colored bar). Proteins were clustered based on dissimilarity measures. The branches are modules of closely correlated proteomic groups that have a similar Log_2FC in late aging and mouse models. Nine significant modules and M0 corresponding to ~1000 genes were detected with WGCNA. M0 is a module with a less correlated protein group. (**b**) Boxplot for log2FC in 24m vs. 12m, average of aAGE and NDD models for pM1, pM2, pM5, pM6, pM7, pM8, and pM9 modules. (**c**) Boxplots of normalized protein-total levels in 6m, 12m and 24m mouse brain, grouped by their modules detected in the WGNCA method. P-values for paired t-test followed by Tukey post hoc test. P-value * ≤ 0.05, ** ≤ 0.01, *** ≤ 0.001 and **** ≤ 0.0001.

bioRxiv preprint doi: https://doi.org/10.1101/2025.08.14.669896; this version posted August 19, 2025. The copyright holder for this preprint (which was not certified by peer review) is the author/funder, who has granted bioRxiv a license to display the preprint in perpetuity. It is made available under aCC-BY 4.0 International license.

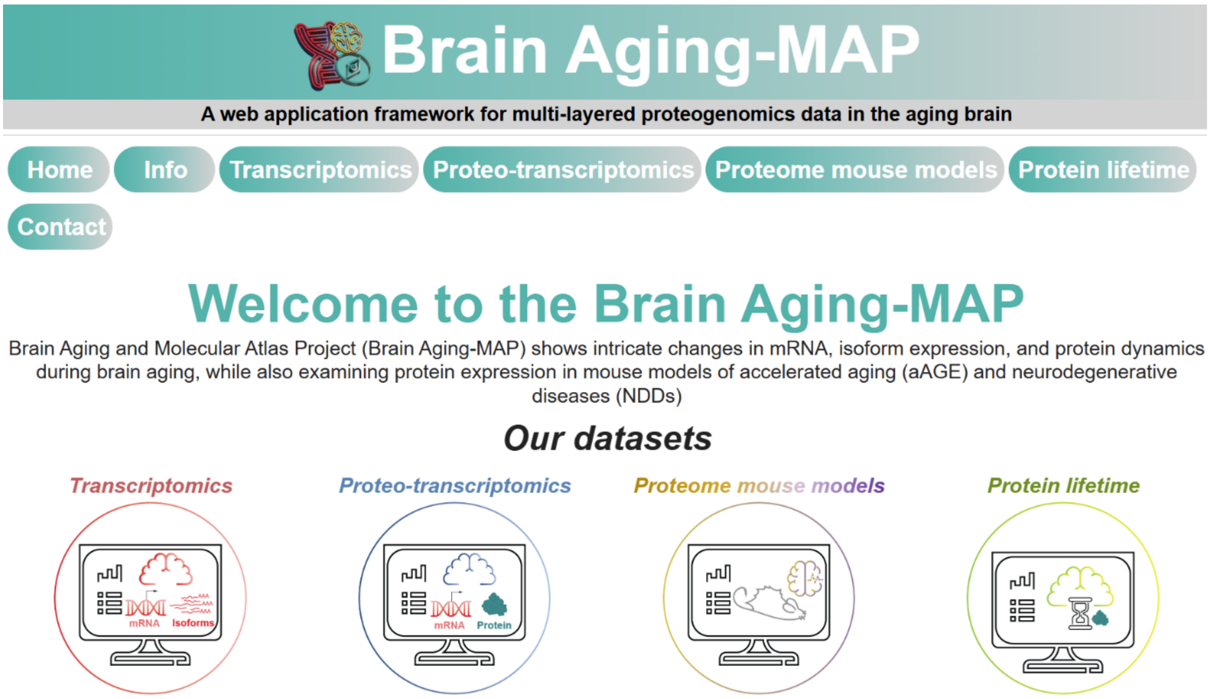

Extended Data Figure 33: First page of the Brain Aging-MAP atlas. This page contains the navigation links to the individual datasets.

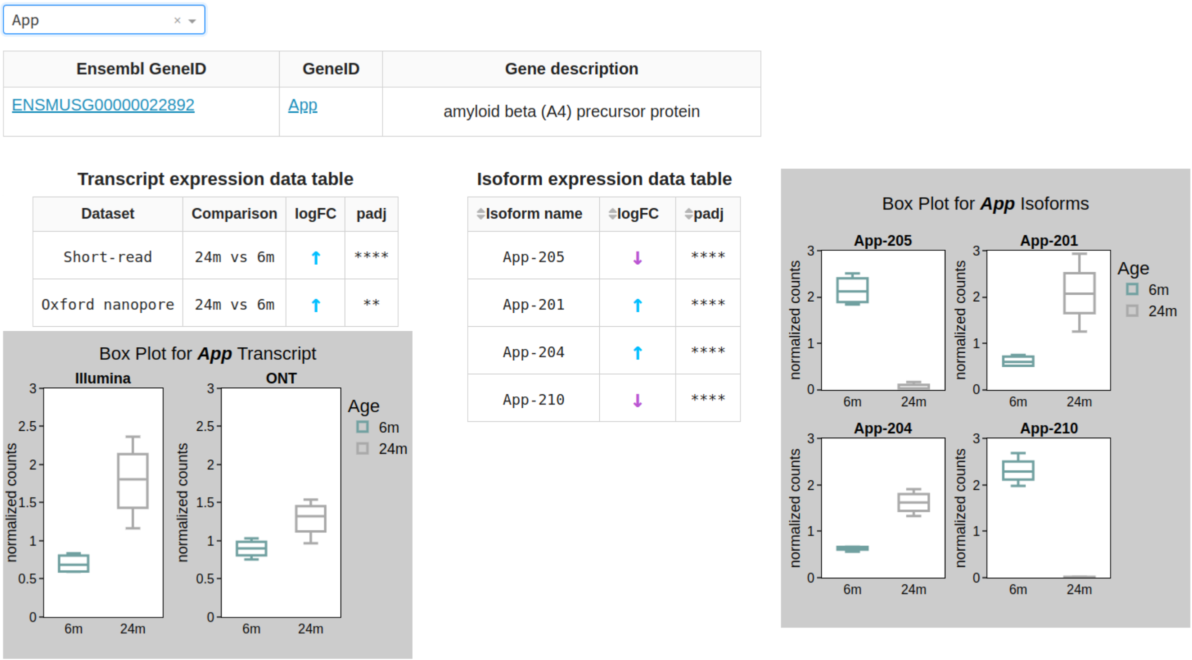

Extended Data Figure 34: Exemplary transcriptomics page of the Brain Aging-MAP atlas. This page contains the gene and isoform expression information in physiological aging.

bioRxiv preprint doi: https://doi.org/10.1101/2025.08.14.669896; this version posted August 19, 2025. The copyright holder for this preprint (which was not certified by peer review) is the author/funder, who has granted bioRxiv a license to display the preprint in perpetuity. It is made available under aCC-BY 4.0 International license.

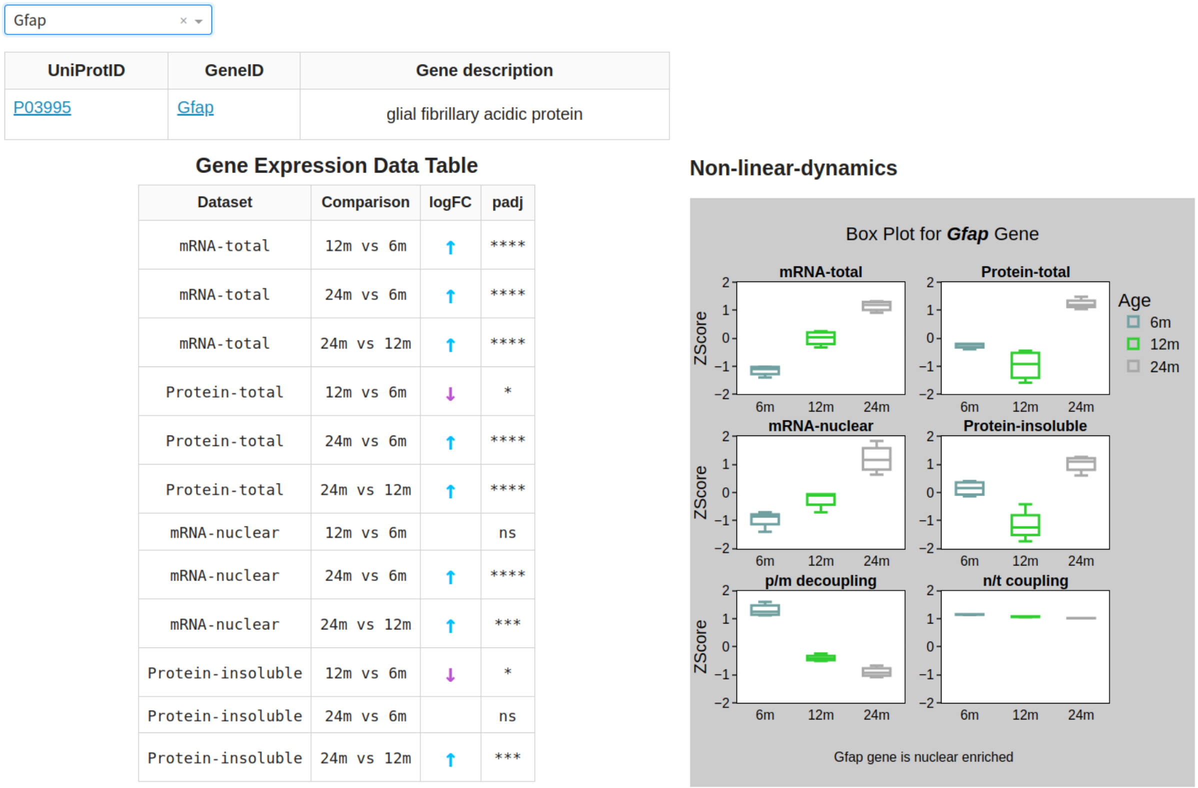

Extended Data Figure 35: Exemplary proteo-transcriptomics page of the Brain Aging-MAP atlas. This page contains the multi-layered proteo-transcriptomics datasets, including total mRNA, nuclear mRNA, total protein, and insoluble protein datasets in physiological aging.

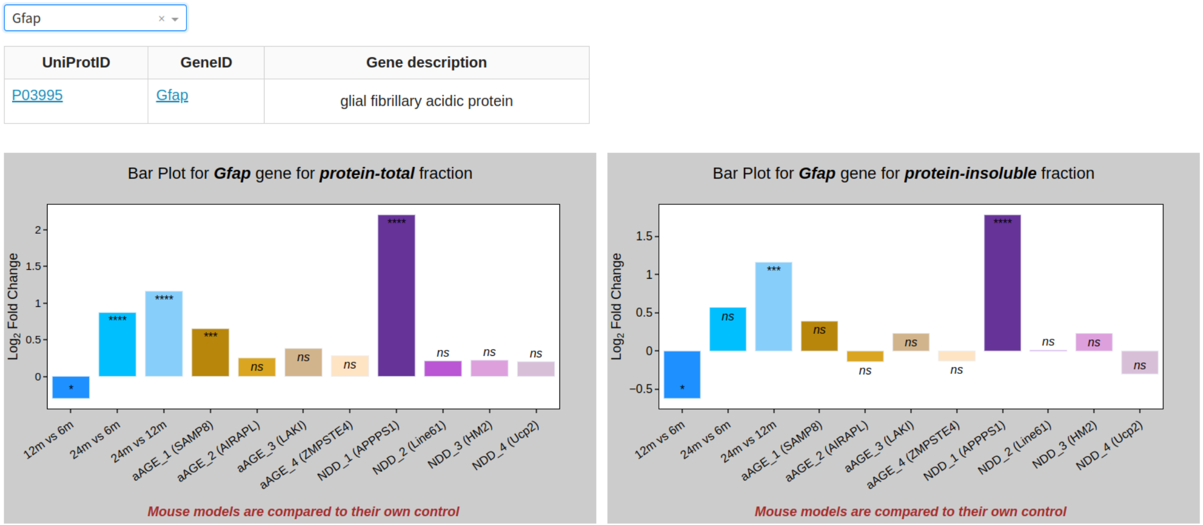

Extended Data Figure 36: Exemplary proteome mouse models page of the Brain Aging-MAP atlas. This page contains the protein expression for the aAGE and NDD mouse models.

bioRxiv preprint doi: https://doi.org/10.1101/2025.08.14.669896; this version posted August 19, 2025. The copyright holder for this preprint (which was not certified by peer review) is the author/funder, who has granted bioRxiv a license to display the preprint in perpetuity. It is made available under aCC-BY 4.0 International license.

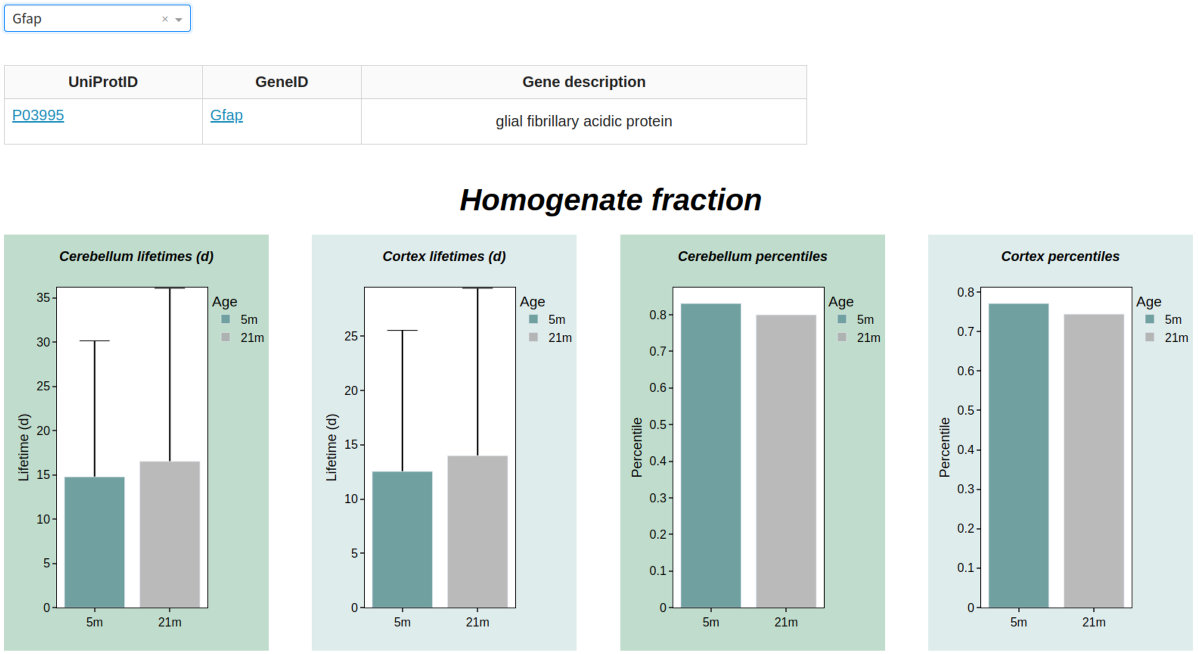

Extended Data Figure 37: Exemplary protein lifetime page of the Brain Aging-MAP atlas. This page contains information about protein lifetime in physiological aging.

bioRxiv preprint doi: https://doi.org/10.1101/2025.08.14.669896; this version posted August 19, 2025. The copyright holder for this preprint (which was not certified by peer review) is the author/funder, who has granted bioRxiv a license to display the preprint in perpetuity. It is made available under aCC-BY 4.0 International license.

Supplementary Text

Supplementary Text 1: mRNA and protein level changes in the total fraction

Differential gene expression analysis in the aging brain was performed by comparing mRNA and protein levels across ages obtained through short-read sequencing and mass spectrometry methods.

We first evaluated genes that exhibited significant differences between 12m and 6m mice (early comparison or early aging) in our mRNA total fraction dataset, with a multiple comparison adjusted p-value (*padj*) ≤ 0.05 and an absolute $\log_2$ fold change ($|\log_2 FC|$) ≥ 0.58 (**Supplementary Table 1**). When considering this subgroup of significantly differentially expressed mRNAs, we found 4184 upregulated transcripts corresponding to ~55% and 3471 downregulated transcripts corresponding to ~45%; **Extended Data Fig. 2a**, and **Supplementary Table 1**).

We then evaluated genes that exhibited significant differences between 24m and 6m mice (overall comparison or overall aging)[24], selecting genes with a *padj* ≤ 0.05 and a $|\log_2 FC|$ ≥ 0.58 (**Supplementary Table 1**). When considering this subgroup of significantly differentially expressed mRNAs, we found 3989 upregulated transcripts corresponding to ~54% and 3394 downregulated transcripts corresponding to ~46% (**Extended Data Fig. 3a**, and **Supplementary Table 1**).

Overall, in 12m vs. 6m and 24m vs. 6m we found a slight over-representation of upregulated genes when compared to 6m. This small bias seems to be due to a bona-fide higher expression rather than a problem of normalization as observed in our previous studies[24]. When analyzing the highest and lowest 50 most significantly changed transcripts in 12m vs. 6m and 24m vs. 6m (**Extended Data Fig. 2b, c and 3b, c**, and **Supplementary Table 1**), we found some interesting hits that include Scgn and Rps29 downregulated in the aged brain. Scgn is a secreted calcium sensor that has a function related to neuroendocrine cells and has recently been shown to interfere with α-synuclein fibrillation [112]. Rps29 is a ribosomal protein that is part of the small ribosomal subunit and is involved in translation to proteins. Among the 50 most upregulated mRNAs we observed a clear enrichment of transcripts encoding proteins with essential neuronal and synaptic functions[24] (**Extended Data Fig. 2c, 3c**, and **Supplementary Table 1**). These include, for example, mRNAs related to the Gamma-Aminobutyric Acid (GABA) transporter (Slc2a13, and Slc7a14), potassium channels (Kcna2, and Kcnd3), and nuclear receptors (Nr2c2, Nr3c1). Other interesting hits include the ALF Transcription Elongation Factor 2 (Aff2) a RNA binding protein that could play a role in alternative splicing and the Neuralized E3 Ubiquitin Protein Ligase 1B (Neurl1b) that has a function related to ubiquitin-dependent endocytosis.

A more wide-ranging gene ontology ORA based on all changed transcripts confirmed a significant downregulation of mitochondrial and ribosomal mRNAs in the aged brain including several gene ontologies (GOs) associated to these processes (e.g., GO:0033108; GO:0007005, GO:0032981, GO:0042254, **Extended Data Fig. 2d, 3d**). The upregulated GOs point to synaptic transmission, organization, dendrite morphogenesis and development (GO:0050804, GO:0048813, GO:0016358, **Extended Data Fig. 2e, 3e**). These results indicate a possible engagement of neurons to produce more synaptic transcripts in the aged brain as observed in our previous study[24].

Furthermore, we evaluated genes that exhibited significant differences between 24m and 12m mice (late comparisons or late aging), with a *padj* ≤ 0.05 and a $|\log_2 FC|$ ≥ 0.58 (**Supplementary Table 1**). Among these significantly differentially expressed mRNAs, we found 64 upregulated transcripts, corresponding to ~39%, and 100 downregulated transcripts,

bioRxiv preprint doi: https://doi.org/10.1101/2025.08.14.669896; this version posted August 19, 2025. The copyright holder for this preprint (which was not certified by peer review) is the author/funder, who has granted bioRxiv a license to display the preprint in perpetuity. It is made available under aCC-BY 4.0 International license.

corresponding to ~61% (**Extended Data Fig. 4a, and Supplementary Table 1**). We observed a slight over-representation of downregulated genes. Notably, the 50 most downregulated transcripts were enriched for neuronal genes, including Shank1, Shank3, Gabrb2, Grm5, and Dlgap3. Additionally, we observed a downregulation of histone genes which play a central role in chromatin remodeling and transcription regulation[113]. These mRNAs include the histone H2, H3, and H4 families, for example, H2ac11, H3c13, H3c15, H4c1, H4c2, H4c3, H4c8, H4c9, H4c11, and H4c12. Interestingly, we also observed a downregulation with aging in two other comparisons: 12m vs 6m and 24m vs 6m (**Extended Data Fig. 3b, Supplementary Table 1).**

Among the 50 most upregulated mRNAs in the 24m vs 12m comparison, we observed genes related to the immune response, which is a hallmark of aging[39–41]. The ORA based on all significantly changed transcripts confirmed a significant downregulation of nucleosome and postsynaptic mRNAs in the aged brain, including several GOs associated with these processes (e.g., GO:0006334, GO:0097107; **Extended Data Fig. 4d**). The upregulated GOs point to the innate immune response and response to interferon beta and gamma (GO:0045087, GO:0034341, GO:0035456, **Extended Data Fig. 4e**). Our analysis revealed a significant upregulation of inflammatory and immune-related genes in the aging brain, consistent with previous reports of neuroinflammation as a hallmark of brain aging[39–41,72] . This immune activation may contribute to synaptic degeneration and functional impairment observed in both normal brain aging and neurodegenerative conditions. Notably, we found that these same pathways, when properly regulated, play crucial roles in neuroplasticity and neuronal stress resistance[39,114]. These findings suggest a delicate balance between beneficial and detrimental effects of immune activation in the aging brain, highlighting the complex interplay between inflammation and cognitive function.

We next analyzed proteins that exhibited significant differences between 12m and 6m mice focusing on proteins in our protein-total fraction dataset using with a *padj* ≤ 0.05 and a $|\log_2 FC| > 0$ (**Supplementary Table 1**). In this comparison, we identified 12 upregulated proteins (~44%) and 15 downregulated proteins (~56%) (**Extended Data Fig. 5a, and Supplementary Table 1**). Similarly, we evaluated proteins with significant differences between 24m and 6m mice, with the same criteria of *padj* ≤ 0.05 and a $|\log_2 FC| > 0$ (**Supplementary Table 1**). Among this subset of significantly differentially expressed proteins, we observed a slight over-representation of upregulated proteins, with 24 upregulated (~77%) and 7 downregulated (~23%) (**Extended Data Fig. 6a, and Supplementary Table 1**). Additionally, when comparing 24m and 12m mice, we found 20 upregulated proteins (~54%) and 17 downregulated proteins (~46%) (**Extended Data Fig. 7a, and Supplementary Table 1**).

We then evaluated genes that exhibited significant changes in mRNA and protein levels and observed an upregulation of astrocyte-specific markers. Glial fibrillary acidic protein (Gfap) often used as an astrocyte marker, showed the most pronounced upregulation, with increased expression at both mRNA and protein levels in 24m mice compared to 6m and 12m mice (**Extended Data Fig. 8, 9, 10 and 11b, c**). Similarly, another astrocyte-specific gene, vimentin (Vim), exhibited elevated expression at 24m compared to 12m (**Extended Data Fig. 10, 11c**) at both transcriptional and translational levels. These results are similar to previous studies[27,39–41,115,116] which suggest glial activation in the aging brain.

Among the proteins significantly downregulated at 24m compared to 6m, we identified TGF-Beta Activated Kinase 1 Binding Protein 3 (Tab3), associated with neuronal apoptosis[117], and Cullin1 (Cul1), involved in proteolysis[118]. These findings suggest alterations in homeostatic processes in the aging brain. We also observed an upregulation of Complement C1q B Chain (C1qb), which is involved in immune function at 24m compared to 6m.

bioRxiv preprint doi: https://doi.org/10.1101/2025.08.14.669896; this version posted August 19, 2025. The copyright holder for this preprint (which was not certified by peer review) is the author/funder, who has granted bioRxiv a license to display the preprint in perpetuity. It is made available under aCC-BY 4.0 International license.

Mitochondrial Contact Site and Cristae Organizing System Subunit 10 (Micos10), involved in inner mitochondrial membrane organization was upregulated at 24m compared to 6m and at 12m compared to 6m. Interestingly, Regulatory Associated Protein of mTOR Complex 1 (Rptor), a component of the mTORC1 complex that regulates cell growth and metabolism was upregulated at 24m compared to 6m at both mRNA and protein levels **(Extended Data Fig 9, 11b)**. This suggests an impaired autophagy and altered protein synthesis potentially due to a hyperactivation of the mTOR pathway[119] (**Extended Data Fig 9, 11b**). This upregulation of mTOR pathway is also associated with neurodegenerative diseases which might suggest an age-related cognitive decline.

ORA of the top 150 proteins across different age comparisons with a non-adjusted p-value < 0.05 revealed distinct of enrichment GO terms (**Supplementary Table 1).** In the early comparison, 'oxoacid metabolic process' (GO:0043436) and 'carboxylic acid metabolic process' (GO:0019752) were significantly enriched among upregulated proteins (**Extended Data Fig 5e**), while 'intermediate filament-based process' (GO:0045103) was enriched among downregulated proteins (**Extended Data Fig 5d**). The late comparison showed a shift, with 'intermediate filament-based process' (GO:0045103, **Extended Data Fig 7e**) becoming enriched among upregulated proteins, and several metabolic processes, including 'monocarboxylic acid metabolic process' (GO:0032787), 'ATP metabolic process' (GO:0046034), and 'oxoacid metabolic process' (GO:0043436), enriched among downregulated proteins (**Extended Data Fig 7d**). The overall comparison showed an activation of GO terms related to immune response (GO:0006956, GO:0002455, **Extended Data Fig 6d**).

Interestingly, several intermediate filaments, including neurofilament polypeptides (Nefl, Nefm, Nefh), vimentin (Vim), plectin (Plec), and α-internexin (Ina), exhibited decreased levels at 12m and were significant in both early and late comparisons (**Extended Data Fig 10, 11a, c**), though not between 24m vs. 6m. This suggests that aging is a non-linear process and highlights the importance of looking more closely at the genes that show a similar trend and the other non-linear changes in the aging process. When analyzing the downregulated proteins at 24m compared to 12m, we found proteins involved in protein folding and stress response such as heat shock proteins Hsp90ab1, Hspa4, and Hsph1, and co-chaperones Ahsa1, Ppid, and Canx (**Extended Data Fig. 1c**).

Furthermore, having perfectly-matched datasets allowed us to perform novel isoform discovery. After combining information from transcriptomics and proteomics, we were able to identify novel isoforms expressed at different ages. We identified several proteoforms (**Supplementary Table 1**) and a notable finding are the two non-canonical sequences of Syt7. Interestingly, the Syt7_1 proteoform, which had 84.1% similarity to the Syt_7 canonical sequence, and this proteoform were significantly downregulated in late aging. From these results, we could speculate that in general, Syt7 protein is downregulated with aging. In the future, having longer reads at 24m and 12m for the transcriptomics datasets could potentially help in the identification of novel isoforms. As these were quantified using the novel isoforms predicted from the 24m and 6m datasets[24].

Supplementary Text 2: mRNA level changes in the nuclear fraction

We then evaluated genes that exhibited significant differences between 12m and 6m mice, in our mRNA-nuclear fraction dataset with a *padj* ≤ 0.05 and a $|\log_2 FC| \geq 0.58$ (**Supplementary Table 5**). This comparison yielded 160 upregulated (~47%) and 189 downregulated (~53%) transcripts (**Extended Data Fig. 21a** and **Supplementary Table 5**). Similar criteria were applied to compare 24m and 6m mice, resulting in 174 upregulated

bioRxiv preprint doi: https://doi.org/10.1101/2025.08.14.669896; this version posted August 19, 2025. The copyright holder for this preprint (which was not certified by peer review) is the author/funder, who has granted bioRxiv a license to display the preprint in perpetuity. It is made available under aCC-BY 4.0 International license.

(~39%) and 204 downregulated (~61%) transcripts (**Extended Data Fig. 22a and Supplementary Table 5**).

A slight over-representation of downregulated genes was observed at 24m and 12m when compared to 6m. Analysis of the 50 most significantly downregulated transcripts in both comparisons (**Extended Data Fig. 21b, 22b and Supplementary Table 5**) revealed notable genes including myosin light chain kinase (Mylk) and myosin light chain 9 (Myl9), which are localized in actin filaments (GO:0032432, GO:0097517; **Extended Data Fig. 21d, 22d and Supplementary Table 5**). Among upregulated genes, immune response-related transcripts were prominent in the 24m vs. 6m comparison (**Extended Data Fig. 22c, e and Supplementary Table 5**), while neuronal mRNAs were more affected in the 12m vs. 6m comparison (GO:0007411, GO:0097485; **Extended Data Fig. 21c, e and Supplementary Table 5**).

Comparison between 24m and 12m mice revealed 48 downregulated (~55%) and 40 upregulated (~45%) transcripts (**Extended Data Fig. 23a and Supplementary Table 5**). Analysis of the most significantly altered transcripts revealed upregulation of genes involved in immune and defense response (GO:0006955**, Extended Data Fig. 23c, e, Supplementary Table 5**) and endopeptidase regulator activity (GO:0004866**; Extended Data Fig. 23c, e; and Supplementary Table 5**) in the aged brain. These included complement C3 (C3), serpin family A member 3 (Serpina3n), bone marrow stromal cell antigen 2 (Bst2), and NLR family apoptosis inhibitory protein (Naip2). Increased levels of Serpina3n and C3 have been associated with age-related changes in brain function and the onset of brain amyloidosis in Alzheimer's disease[120,121] (AD). Notably, Ran-Binding Protein 6 (RanBP6), a nuclear transport receptor, was downregulated in both 24m vs. 6m and 12m vs. 6m comparisons, aligning with known nuclear transport dysfunction in the aged brain[122]. Additionally, we observed significant downregulation of synapse and calcium ion binding mRNAs in the aged brain for 24m vs 12m comparison (GO:0045202; **Extended Data Fig. 23b, d; and Supplementary Table 5**).

Overall, our analysis in the physiologically aging brain revealed a downregulation of genes involved in synapse function and an upregulation of immune response in the late comparisons, consistent with the previous studies[39–41].

Supplementary Text 3: Age-related changes in protein solubility

An upregulation at 24m for some proteins involved in cell signaling, trafficking, protein translation, and synaptic maintenance. For instance, Iqsec2, Anxa11, Anxa6 and Rpl10. Notably, we found that Iqsec2 has an IUPRED score of 0.7, indicating that it has a high amount of intrinsically disordered regions. Interestingly, 60S ribosomal proteins showed an increased insolubility at 24m (**Fig. 4e**) suggesting a reduction in functional ribosomes in the cytoplasm. However, we did not observe significant differences in the insolubility for the cytoskeletal, mitochondrial and proteasomal proteins (**Fig. 4d; Extended Data Fig. 27**).

Supplementary Text 4: Age-related shifts in metabolic costs

We calculated the metabolic cost per proteo-transcriptome species based on protein turnover parameters previously measured in young and aged brains in a previous work[5] and combined mRNA and protein level measurements obtained in this study. We found that the distribution of cost per proteo-transcriptome species is heavy-tailed, spans roughly four orders of magnitude and is superimposable at different ages (**Extended Data Fig. 29a**). When comparing the cost change in aged brains vs young ones, we found that specifically high-cost proteins experience a reduction in their energetic burden with age (**Extended Data Fig. 29a**), leading to a proteome-wide cost reduction of 26.24%. In contrast, proteins with low cost

bioRxiv preprint doi: https://doi.org/10.1101/2025.08.14.669896; this version posted August 19, 2025. The copyright holder for this preprint (which was not certified by peer review) is the author/funder, who has granted bioRxiv a license to display the preprint in perpetuity. It is made available under aCC-BY 4.0 International license.

exhibited elevated energetic demands with age. Neither mitochondrial, ribosomal, nor synaptic proteins show strongly deviating trends from the overall background (**Extended Data Fig. 29b)** Overall, the changes in costs are driven by both variable parameters, protein half-life and protein levels (**Extended Data Fig. 29c, d**). If reduction in energy availability requires maintaining abundant but expensive proteins under increased translational pressure, this would lead to an increased translational efficiency over proteins with a low energetic burden, leading to a relative increase in their levels despite high biosynthetic cost. This rebalancing of proteome composition in aging is possibly an adaptive reaction to maintain cellular homeostasis during the condition of limited metabolic resources.

Supplementary Text 5: Protein level changes in the mouse models

To obtain the proteins that are differentially expressed, we performed differential gene expression analysis and selected genes that showed significant changes (*padj* ≤ 0.05) in at least one mouse model or in physiological aging, in either protein total or insoluble fraction (**Supplementary Table 8**). This resulted in ~1000 genes for our further analysis that includes multiomics gene set enrichment analysis (GSEA) and WGCNA analysis. Among the genes that were differentially expressed we observed significant changes for the genes involved in gliosis, inflammation, maintenance of neuronal structure, protein modification pathways, protein homeostasis, and lipid metabolism. For example, Gliosis and inflammation markers Gfap and Vim (**Extended Data Fig. 31a, b**), were upregulated in late aging (24m vs 12m), several aAGE models, and NDD models. Plectin (Plec, **Extended Data Fig. 31c**), involved in maintenance of cytoskeletal structure, was upregulated in late aging, all NDD models, and two aAGE models, but downregulated in aAGE_3. SUMO1 Activating Enzyme Subunit 1 (Sae1, **Extended Data Fig. 31d**) was downregulated in late aging and several models but upregulated in NDD_2. Glycerol-3-phosphate dehydrogenase (Gpd2, **Extended Data Fig. 31e**) was upregulated in late aging and all models, particularly significant in aAGE models and NDD_2. Chaperone regulation was impacted, as evidenced by the strong downregulation of Hsp90ab1 (**Extended Data Fig. 31f**) in late aging and nuclear envelope-related aAGE models. Oxysterol Binding Protein Like 1A (Osbpl1a, **Extended Data Fig. 31g**), gene involved in lipid metabolism was downregulated for late aging and all mouse models except aAGE_2 and NDD_4, which showed an upregulation. Succinyl-CoA:3-ketoacid coenzyme A transferase 1, mitochondrial (Oxct1, **Extended Data Fig. 31h**), involved in ketone body metabolism was significantly upregulated in three aAGE models but downregulated in NDD_3. Leucine Aminopeptidase 3 (Lap3, **Extended Data Fig. 31i**) was significantly upregulated in late aging and two NDD models but downregulated in nuclear envelope-related aAGE models (aAGE_3 and aAGE_4).
